# Identification of the receptor of oncolytic virus M1 as a therapeutic predictor for multiple solid tumors

**DOI:** 10.1038/s41392-022-00921-3

**Published:** 2022-04-08

**Authors:** Deli Song, Xudong Jia, Xincheng Liu, Linyi Hu, Kaiying Lin, Tong Xiao, Yangyang Qiao, Jiayu Zhang, Jia Dan, Chunwa Wong, Cheng Hu, Ke Sai, Shoufang Gong, Max Sander, Runling Shen, Xiaoyu Chen, Xiaoting Xiao, Jiehong Chen, Yanming Zhang, Cailv Wei, Xiao Xiao, Jiankai Liang, Qinfen Zhang, Jun Hu, Wenbo Zhu, Guangmei Yan, Yuan Lin, Jing Cai

**Affiliations:** 1 Department of Pharmacology, Zhongshan School of Medicine, Sun Yat-sen University, Guangzhou, 510080 China; 2 School of Life Sciences, Sun Yat-sen University, Guangzhou, 510275 China; 3 Department of Urology, The Third Affiliated Hospital of Sun Yat-sen University, Guangzhou, 510630 China; 4 Department of Neurosurgery/Neuro-oncology, Sun Yat-sen University Cancer Center, Guangzhou, 510060 China; 5 Guangzhou Virotech Pharmaceutical Co. Ltd, Guangzhou, 510663 China; 6 Department of Microbiology, Zhongshan School of Medicine, Sun Yat-sen University, 510080 Guangzhou, China

**Keywords:** Cancer therapy, Predictive markers, Target identification, Predictive markers, Cancer therapy

## Abstract

Over the last decade, oncolytic virus (OV) therapy has shown its promising potential in tumor treatment. The fact that not every patient can benefit from it highlights the importance for defining biomarkers that help predict patients’ responses. As particular self-amplifying biotherapeutics, the anti-tumor effects of OVs are highly dependent on the host factors for viral infection and replication. By using weighted gene co-expression network analysis (WGCNA), we found matrix remodeling associated 8 (MXRA8) is positively correlated with the oncolysis induced by oncolytic virus M1 (OVM). Consistently, MXRA8 promotes the oncolytic efficacy of OVM in vitro and in vivo. Moreover, the interaction of MXRA8 and OVM studied by single-particle cryo-electron microscopy (cryo-EM) showed that MXRA8 directly binds to this virus. Therefore, MXRA8 acts as the entry receptor of OVM. Pan-cancer analysis showed that MXRA8 is abundant in most solid tumors and is highly expressed in tumor tissues compared with adjacent normal ones. Further study in cancer cell lines and patient-derived tumor tissues revealed that the tumor selectivity of OVM is predominantly determined by a combinational effect of the cell membrane receptor MXRA8 and the intracellular factor, zinc-finger antiviral protein (ZAP). Taken together, our study may provide a novel dual-biomarker for precision medicine in OVM therapy.

## Introduction

Oncolytic virus (OV) is a kind of replication-competent virus that preferentially recognizes and replicates in various tumor cells without harming normal tissues^1^. OV therapy exhibits an optimistic clinical potential in terms of its particular dual mechanisms: (i) direct oncolysis and (ii) activation of antitumor immune response^1,2^. Since Talimogene laherparepvec (T-vec), the first OV medicine approved by U.S. Food and Drug Administration, achieved encouraging success in melanoma, an increasing number of OVs have been moving toward clinical use^3,4^.

Although OV therapy has put a ray of light for cancer treatment and dramatically improved outcomes in a part of patients with some refractory tumors, such as glioma and advanced-stage melanoma, a large portion of patients could not yet effectively benefit from it within a “responsive” tumor type^4,5^. One of the pivotal reasons is that most of the OVs in the clinical stage lack effective predictive biomarkers, which are able to distinguish responders and non-responders among all patients. Therefore, identifying and understanding the molecular mechanisms which underlie the selectivity of OVs and can be used as predictive biomarkers are critical for the further development of the whole field of OV medicines.

Unlike the drugs of chemotherapy and protein-based therapies, OVs are the unique self-amplifying medicines that can replicate and spread sufficiently in the tumor^6^. In this case, the cellular membrane viral receptor and the intracellular host factors have determinant effects on viral infection and replication. The complex genetic heterogeneity in human tumors could lead to a huge variety of these host determinants even among patients with cancer of the same histological type^7^.

In the present study, we firstly identified that matrix remodeling associated 8 (MXRA8)^8^ is ubiquitously expressed on multiple solid tumor tissues and functions as the receptor for oncolytic virus M1 (OVM), a novel OV identified by our group^9–13^. Furthermore, we probed insight into the tumor selectivity of the OVM and offered a rationale for defining the expression of MXRA8 and zinc-finger antiviral protein (ZAP) in tumors as a potential dual-biomarker for OV precision medicine in future clinical use.

## Results

### Identification of MXRA8 as a key factor in oncolysis induced by oncolytic virus M1

**Fig. 1:**
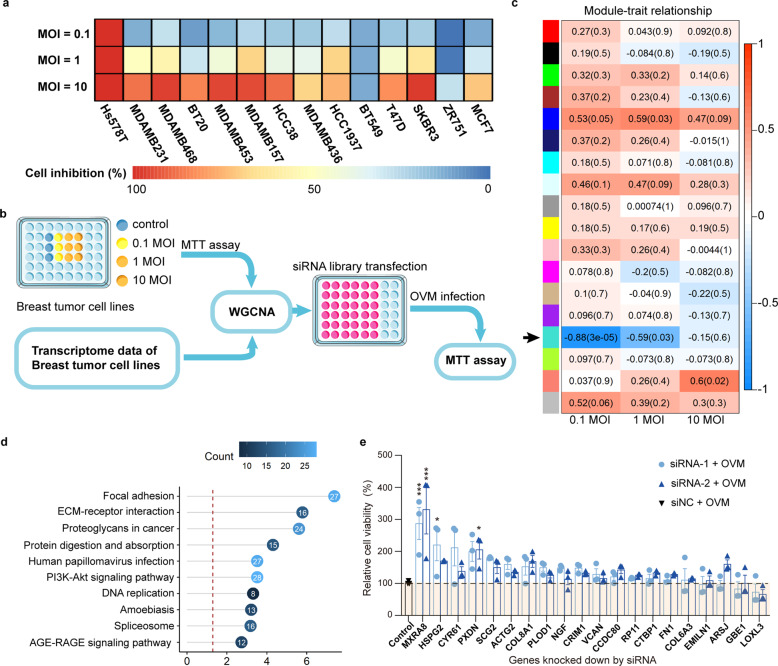
The therapeutic efficacy of OVM is related to the tumor expression level of MXRA8. **a** Cell inhibition rates in 14 breast tumor cell lines treated with OVM (MOI = 0.1, 1, or 10) for 72 h. **b** An outline of analysis protocol. Firstly, tumor cells were seeded in 48-well plates and treated with OVM in respective MOI for 72 h and detected by MTT. Secondly, transcriptome data of 14 breast tumor cell lines were obtained from Broad Institute Cancer Cell Line Encyclopedia (CCLE) and analyzed by WGCNA. Lastly, secondary screen was further conducted to identify genes associated with OVM-induced oncolysis. **c** Spearman’s correlation coefficient between the co-expression modules (shown as the colored boxes) and the cell viability in escalating MOI of OVM analyzed by WGCNA. **d** The major pathways enriched in genes of turquoise co-expression module determined by KEGG analysis. **e** Hs578T cells transfected with siRNAs targeting the top 20 differentially expressed genes were incubated with OVM (0.1 MOI) and analyzed for cell viability by MTT (three experiments, *n* = 3; one-way ANOVA; mean ± s.d.). *P < 0.05; **P < 0.01; ***P < 0.001

To assess the oncolytic efficiency of OVM, we treated 14 breast tumor cell lines with escalating doses of OVM (MOI = 0.1, 1, or 10). Although OVM showed an encouraging oncolytic effect in most breast tumor cell lines at 10 MOI, the sensitivity to OVM within tested cell lines varied greatly (Fig. 1a; for example, 96% inhibition rate at 0.1 MOI in Hs578T cell line; 2% inhibition rate at 10 MOI in BT549 cell line). The huge difference in tumor cell oncolysis within one type of tumor revealed a challenge for OVM therapy posed by tumor heterogeneity. Moreover, we found the different levels of sensitivity to OVM in these cell lines had no obvious correlation with ZAP, endoplasmic reticulum to nucleus signaling 1 (ERN1), and ras homolog family member (RHOQ) expression, which are associated with OVM viral replication (Supplementary Fig. 1a–c)^9,12,14^. The diverse responses to OVM indicated the existence of other factors that modulate OVM’s tumor selectivity.

By calculating the Pearson correlation coefficient of the 3718 most varying genes in 14 breast tumor cell lines, we clustered genes into several co-expression modules labeled with different colors. To identify the co-expression modules that are significantly associated with oncolysis, modules were compared with cell viability at 0.1, 1, or 10 MOI of OVM treatment using Spearman’s correlation (Fig. 1b, Supplementary Fig. 1d). We identified the turquoise module showing the highest degree of negative correlation with the cell viability (Fig. 1c; 0.1 MOI, *R* = −0.88; 1 MOI, *R* = −0.59). Further analysis demonstrated that genes within the turquoise module were enriched in focal adhesion and ECM-receptor interaction (Fig. 1d). To further identify genes associated with OVM-induced oncolysis, we conducted an RNAi screen to target the top 20 genes in the turquoise module (Supplementary Fig. 1e, Supplementary Table 1). siRNA-transfected Hs578T cells were either mock-infected or infected with OVM at 0.1 MOI and measured for cell viability by MTT assay (Fig. 1b, Supplementary Fig. 1f). Among these genes, transfecting of siRNA targeting matrix remodeling associated 8 (MXRA8, also called limitrin, ASP3 or DICAM), a membrane protein that normally interacts with αvβ integrin, associated with the blood-brain barrier and was recently identified as an entry receptor for chikungunya virus (CHIKV)^8,15,16^, could significantly improve cell viability of Hs578T cells under OVM infection (Fig. 1e). These data indicate that MXRA8 could contribute to the oncolytic effect of OVM in breast tumor cells and plays an essential role in OVM’s life cycle.

### Expression of MXRA8 promotes the therapeutic efficacy of oncolytic virus M1 in vitro and in vivo

**Fig. 2:**
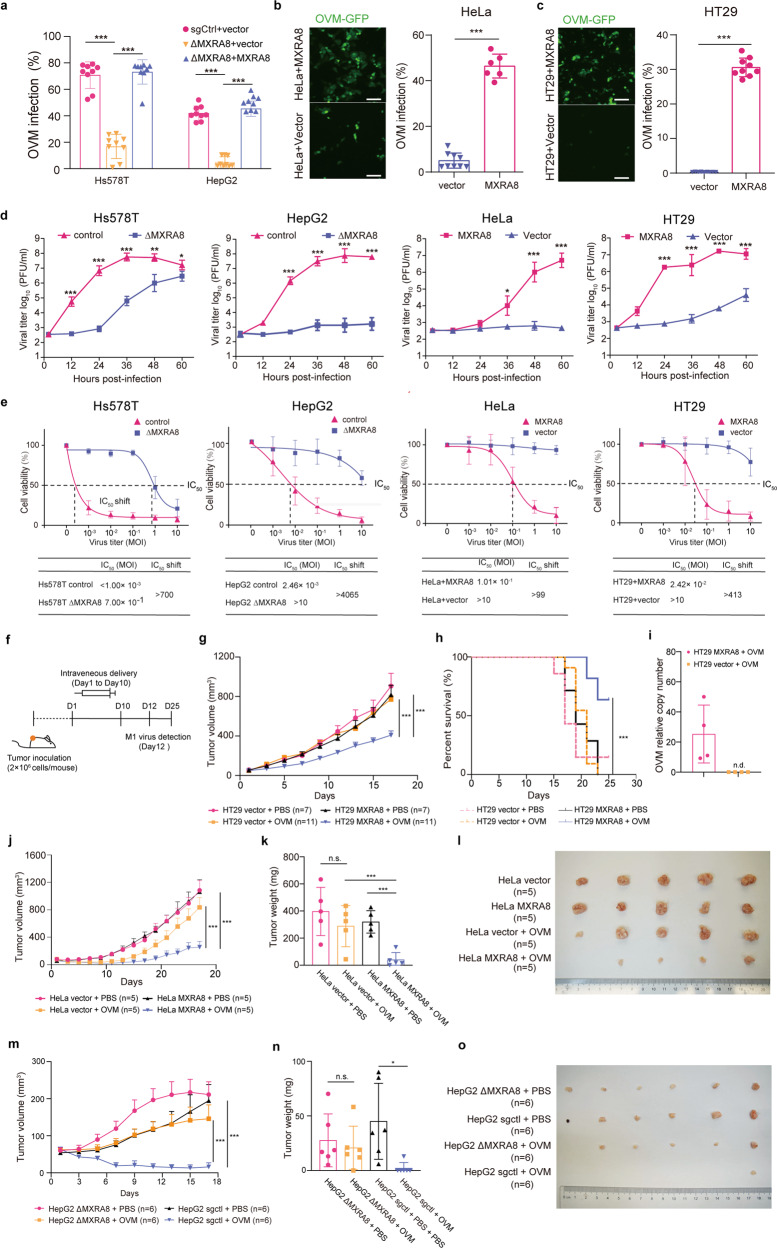
MXRA8 promotes the therapeutic efficacy of OVM in vitro and in vivo. **a** The infection rates of OVM-GFP in control, ΔMXRA8 (sgRNA-1) and ΔMXRA8 (sgRNA-1)+MXRA8 Hs578T or HepG2 cells (MOI of 0.1 for 48 h at 37 °C) by flow cytometry (three experiments, *n* = 9; one-way ANOVA; mean ± s.d.). **b, c** MXRA8-overexpressing cells and control cells were infected with OVM-GFP virus at a multiplicity of infection (MOI) of 1 (HeLa cells, 48 h; HT29 cells, 24 h), after which the phase-contrast and fluorescence microscopy images were captured, and further processed for detection of GFP expression by flow cytometry (three experiments, *n* = 9; one-way ANOVA; mean ± s.d.). Scale bars: 50 μm. **d** The viral titer of tumor cells that treated with OVM at 0.01 MOIs for 72 h by CCID50 assay (three experiments, *n* = 9; one-way ANOVA; mean ± s.d.). **e** The viability of tumor cells treated with OVM at the respective MOIs for 72 h by MTT assay (three experiments, *n* = 9; one-way ANOVA; mean ± s.d.). **f** Timeline of the experimental arrangement for **g**–**o**. **g** Tumor growth curves of the average HT29 tumor volumes in each group. **h** Kaplan–Meier survival curves of HT29 tumor-bearing mice by log-rank test. **i** The amount of OVM in tumor tissues was measured by qPCR at 2 days after the 10th injection (*n* = 4, per group). **j** Tumor growth curves of the average HeLa tumor volumes in each group. **k** HeLa tumor weights on the 27th day after the first OVM treatment (one-way ANOVA; mean ± s.d.). **l** Photograph of HeLa tumor tissues on the 27th day. **m** Tumor growth curves of the average HepG2 tumor volumes in each group. **n** HepG2 tumor weights on the 23rd day after the first OVM treatment (one-way ANOVA; mean ± s.d.). **o** Photograph of HepG2 tumor tissues on the 23rd day. n.d., not detectable. Tumor growth curves were analyzed by repeated-measures ANOVA in a general linear model. *P < 0.05; **P < 0.01; ***P < 0.001

To evaluate whether the promoting effect of MXRA8 for OVM could be a universal phenomenon, we tested several types of tumor cell lines. According to the Cancer Cell Line Encyclopedia (CCLE) database, MXRA8 is highly expressed on HepG2 (hepatocellular carcinoma) and Hs578T (breast carcinoma) cells, but not detectable on HeLa (cervical carcinoma) or HT29 (colorectal carcinoma) cells (Supplementary Fig. 2a). Ectopically expressing MXRA8 on HeLa and HT29 cells resulted in significantly elevated infection rates of OVM, while the infection rate of OVM was largely decreased in ΔMXRA8 Hs578T and HepG2 cells and further restored with MXRA8 trans-complementation (Fig. 2a–c, Supplementary Fig. 2b–d). Moreover, Amplification curves of OVM revealed that the viral replication was increased in HeLa and HT29 cells overexpressing MXRA8, while depletion of MXRA8 reduced viral replication in Hs578T and HepG2 cells (Fig. 2d).

After OVM hijacked the protein synthesis system of infected cells, abnormal accumulation of misfolded proteins leads to endoplasmic reticulum (ER) stress-induced apoptosis^14^. By using transmission electron microscopy, we observed more severe destruction of organelles and increased viral production in HeLa and HT29 cells overexpressing MXRA8 (Supplementary Fig. 2e). Consistently, the promotion of OVM infection and amplification of ER stress proteins, such as pPERK (phospho–protein kinase RNA–like endoplasmic reticulum kinase), and pEIF2A (phospho–eukaryotic initiation factor 2a), on MXRA8 expressing tumor cells led to boosted OVM oncolysis, as the dose required to kill 50% of the cells (IC_50_) was sharply reduced in MXRA8-expressing cells (99-fold in HeLa, 413-fold in HT29, 700-fold in Hs578T and 4065-fold in HepG2 cells) (Fig. 2e, Supplementary Fig. 2f, g).

To assess the impact of MXRA8 on the therapeutic efficacy of OVM therapy in vivo, OVM was delivered intravenously into tumor-bearing mice from the 1st day to the 10th day (Fig. 2f). In the groups of MXRA8-expressing cells, the administration of OVM significantly inhibited tumor growth, while OVM-induced tumor suppression was nearly absent in HT29 cells without MXRA8 expression (Fig. 2g). In addition, a longer survival time was observed in mice bearing MXRA8-expressing HT29 cells treated with intravenous delivery of OVM (Fig. 2h). Moreover, we measured the amount of OVM in the tumor site on the 12th day (2 days after the last virus injection). Unlike the group of mice bearing MXRA8-expressing HT29 tumors treated with OVM, mice bearing vector-expressing HT29 tumors exhibited undetectable level of OVM in tumor tissues (Fig. 2i). Consistent with the result of HT29 cells, OVM reached a better therapeutic efficacy in HeLa and HepG2 tumors with MXRA8 expressing, and the total tumor weight was significantly reduced in MXRA8-expressing group treated with OVM compared to MXRA8-deficient group (Fig. 2j–o). Moreover, mice in all the three tumor-bearing models showed no signs of obvious toxicity or weight loss during OVM treatment (Supplementary Fig. 3a–c).

Overall, our data demonstrated that high MXRA8 expression in tumor cells could promote the therapeutic efficacy of OVM in several tumor types.

### MXRA8 functions as the entry receptor of oncolytic virus M1

To identify which important role that MXRA8 played in OVM’s life cycle, we firstly evaluated whether MXRA8 is required for OVM’s attachment and internalization. The results showed that both relative binding and internalization activity of OVM in MXRA8-expressing cells were significantly increased compared to ones with lower MXRA8 expression (Supplementary Fig. 4a, b). Moreover, pre-incubation with MXRA8 protein significantly reduced OVM infection in MXRA8-expressing cells in a dose-dependent manner (Supplementary Fig. 4c), while not directly affecting the intracellular replication of OVM through viral RNA transfection (Supplementary Fig. 4d).

**Fig. 3:**
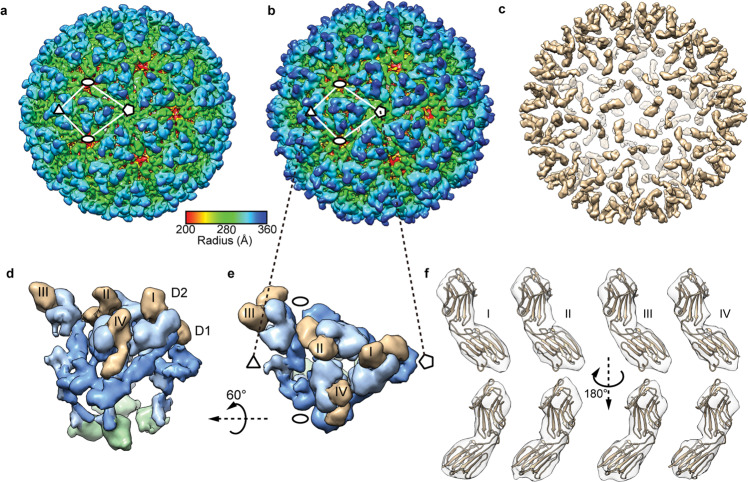
Cryo-EM structures of OVM and the OVM-MXRA8 complex. **a**, **b** Radially colored cryo-EM maps of unbound OVM (**a**) and the OVM-MXRA8 complex (**b**). The locations of the icosahedral five-, three- and two-fold axes are marked with pentagons, triangles, and ellipses, respectively. The bar shows the color scheme. **c** The difference map between unbound OVM and the OVM-MXRA8 complex. To highlight the major difference, small densities were hidden. **d**, **e** Enlarged view of an asymmetric unit of the OVM-MXRA8 complex. **e** is the enlarged view of the boxed asymmetric unit in (**b**). **d** is the asymmetry unit (**e**) rotated by 60°. The E1 protein is colored dark blue. The E2 protein is colored in light blue. The C protein is indicated in green. The density of MXRA8 is shown in tan. The numerals I–IV indicate the densities of four MXRA8 ectodomains. **f** The MXRA8 atomic model (PDB: 6JO8) was docked into the four MXRA8 densities, as viewed from two opposite directions

To obtain direct structural evidence that MXRA8 can be recognized and bound by OVM, we applied single-particle cryo-EM techniques. The cryo-EM structure of OVM and the OVM-MXRA8 complex, at an overall resolution of ~8.3 Å and 12 Å, respectively (Supplementary Fig. 5a, b) were obtained for the first time. The cryo-EM density map of OVM (Fig. 3a) showed *T* = 4 icosahedral symmetry with 240 copies of E2–E1 heterodimers assembled into 80 trimeric spikes. One of the E2 domains was located at the outermost region of the spike, surrounded by the other E1 and E2 domains forming the triangular bottom region of the spike. The outermost spike and triangular platform regions co-organize to form a glycoprotein shell. Under the glycoprotein shell is the lipid bilayer. The transmembrane helixes of E1/E2 extend from the triangular platform and through the lipid layer to the inner nucleocapsid shell (Supplementary Fig. 5c, d). Electron densities inside of the nucleocapsid shells are produced by disordered genomic RNA (Supplementary Fig. 5c, d).

The cryo-EM structure of the OVM-MXRA8 complex showed additional MXRA8 densities around trimeric spikes compared with unbound OVM (Fig. 3a, b). The central cross section (Supplementary Fig. 5e) of the OVM-MXRA8 complex also exhibited the additional MXRA8 densities attached to the spikes. To clearly illustrate the structural differences, we generated a difference map revealing the only MXRA8 densities (Fig. 3c). The MXRA8 binds to E2–E1 heterodimers in a 1:1 ratio, with 240 copies bound per OVM particle, and embeds under the outermost domain of E2. Its density has a dumbbell-like shape, which is in agreement with its X-ray crystal structure^17^. The cross-correlation results (Supplementary Table 2) revealed that domain 2 (D2) extends to the outermost surface of the OVM-MXRA8 complex, and the domain 1 (D1) points towards the interior along the edge of the E1–E2 dimer (Fig. 3d, e). In each asymmetric unit, the four additional densities are very similar but not fully identical to each other (Fig. 3f).

Collectively, all the aforementioned results indicated that MXRA8 binds directly to a specific position on the surface of OVM and acts as the entry receptor of OVM. Although the results showed that >60% of amino acids in the CHIKV-MXRA8 interaction sites are variable in the E2 glycoprotein of OVM (Supplementary Fig. 6), the binding of MXRA8 in OVM infectious particles is in agreement with its binding in CHIKV virus-like particle (VLP), gathering more evidences for MXRA8 being a common receptor among several alphaviruses.

### The heterogenous distribution of MXRA8 in multiple human solid tumors

**Fig. 4:**
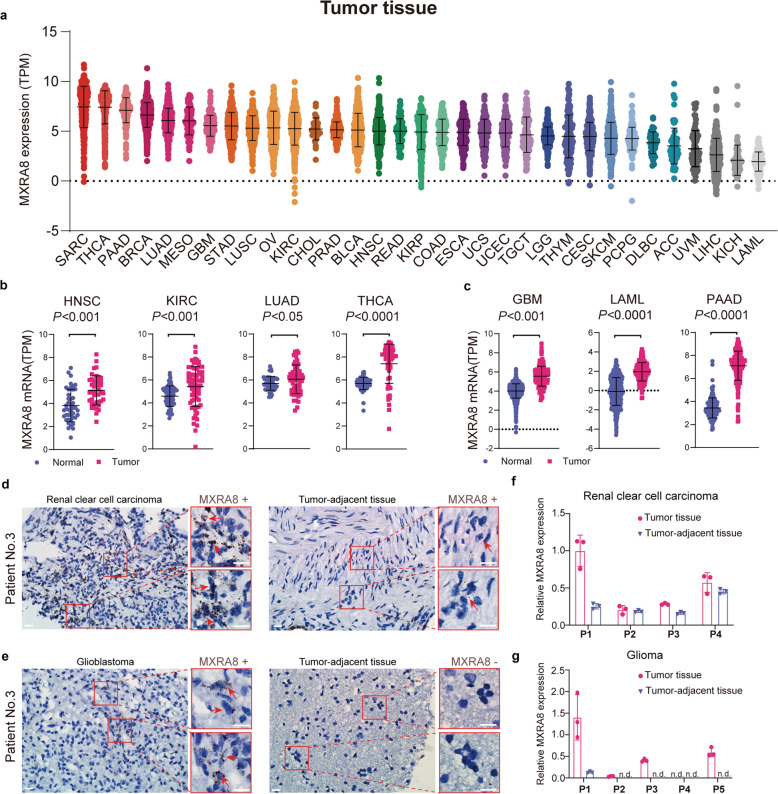
MXRA8 is abundant in human tumor tissues but shows different expression levels among patients. **a** An overview of the RNA-seq data showing the MXRA8 expression levels in human tumor tissues according to the TCGA database. **b** Pan-analysis of the mRNA expression levels of MXRA8 in tumor tissues compared with the corresponding normal tissues in the TCGA database by paired *t*-test (HNSC, *n* = 43; LUAD, *n* = 60; KIRC, *n* = 58; THCA, *n* = 59). Each dot represents a single patient. **c** The expression levels of MXRA8 in tumor tissues from the TCGA database compared with those in the corresponding normal tissues from the TCGA and GTEX databases by two-tailed unpaired *t*-test. (GBM, *n* = 165 tumor versus 1146 normal tissues; PAAD, *n* = 178 versus 165; LAML, *n* = 173 versus 444). **d**, **e** RNA-scope detection of RNA in renal cancer (**d**) and glioma (**e**) with probes against MXRA8. Scale bars: 20 μm. **f**, **g** The expression of MXRA8 in renal cancer and glioma was measured by RT-qPCR. n.d., not detectable; *P < 0.05; **P < 0.01; ***P < 0.001

To evaluate the role of MXRA8 in targeting OVM to tumors, a pan-cancer analysis of RNA sequencing data from the Genotype Tissue Expression Project (GTEx) and The Cancer Genome Atlas (TCGA) was used to examine the expression of MXRA8 in various types of cancers. TCGA data revealed high expression of MXRA8 in nearly all solid tumors (Fig. 4a, Supplementary Table 3). Moreover, MXRA8 was significantly upregulated in head and neck squamous cell carcinoma (HNSC), lung adenocarcinoma (LUAD), kidney renal clear cell carcinoma (KIRC) and thyroid carcinoma (THCA) tissues compared with the corresponding normal tissues as evaluated by a paired *t*-test (Fig. 4b). It was also significantly upregulated in glioblastoma multiforme (GBM), pancreatic adenocarcinoma (PAAD), and acute myeloid leukemia (LAML) compared with normal tissues (Fig. 4c).

To further investigate the distribution and expression of MXRA8 in tumor tissues, RNA-scope, an RNA in situ hybridization technology, was applied to measure MXRA8 expression levels in several renal clear cell carcinoma and glioma patient-derived samples (Fig. 4d, e, Supplementary Fig. 7a, b). Consistent with the pan-cancer analysis results described above, strong positive MXRA8 staining and upregulated MXRA8 expression were detected in most renal tumor tissues and glioma tissues compared to the corresponding tumor-adjacent tissues (Fig. 4f, g). In addition, the promoting effect of MXRA8 was further demonstrated on 769-P (kidney carcinoma) and U-87MG (glioma) cell models (Supplementary Fig. 7c–f). Moreover, to further elucidate the expression of MXRA8 in liver and breast tumors, immunohistochemistry (IHC) was performed on tumor tissue microarrays and normal tissues. The results showed that most of the liver (89.74%, *n* = 90) and all breast (100%, *n* = 39) tumor tissues were observed with MXRA8 positive staining, and MXRA8 in liver tumor is upregulated compared to liver normal tissues (Supplementary Fig. 7g–l).

Although these results demonstrated that MXRA8 was universally expressed on multiple tumor types, such as breast cancer, kidney cancer, and glioma, we found that the level of MXRA8 expression varied greatly among patients even within the same histological type (Fig. 4a, Supplementary Table 3). For example, in 530 patients with KIRC, four patients showed extremely low expression of MXRA8, and the MXRA8 expression ranged from −2.114 to 9.946 Transcripts PerKilobase Million (TPM) (Fig. 4a, Supplementary Table 3). Moreover, in this study, we also observed that one glioma and 23 liver tumor patients were negative for MXRA8 staining by RNA-scope or IHC, and the tumor MXRA8 expression in brain, kidney, breast, and liver tumor patients varied wildly (Fig. 4d–g, Supplementary Fig. 7g–l).

Overall, these evidences showed that MXRA8 is ubiquitously expressed in human solid tumor tissues and selectively upregulated in certain types of cancer, but MXRA8 expression also showed large individual differences among patients. Thus, detection of MXRA8 expression in tumor is an essential step for designing individualized OVM therapy.

### The tumor selectivity of oncolytic virus M1 is concurrently modulated by MXRA8 and ZAP

Although our results had shown a dominant role of MXRA8 in OVM’s oncolysis in several tumor models, interestingly, the regular and even ectopic expression of MXRA8 in normal cells had a negligible impact on the sensitivity to OVM (Supplementary Fig. 8a, b). Moreover, our result showed that MXRA8 overexpression brought limited benefit in the oncolysis of OVM to LoVo cell line (colorectal carcinoma) which is resistant to OVM (Supplementary Fig. 8c–e). All these data indicated that we could not evaluate the tumor response to OVM only by measuring the expression of the viral receptor.

**Fig. 5:**
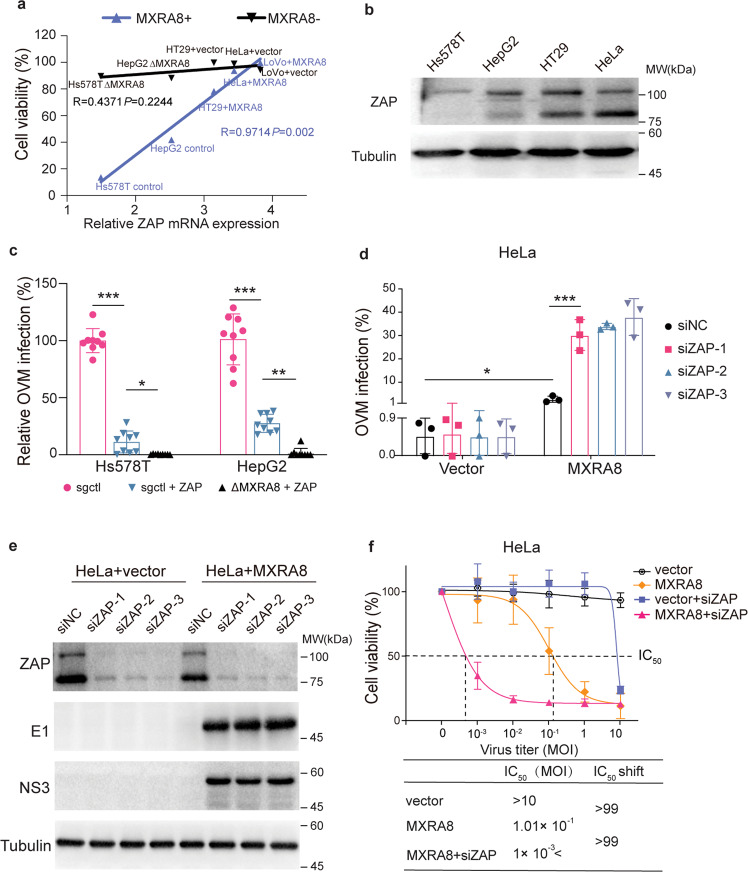
The tumor selectivity of OVM is modulated by a comprehensive effect of MXRA8 and ZAP. **a** Spearman’s correlation coefficient of cell viability under OVM treatment and ZAP expression levels. *R* indicates the Spearman’s correlation coefficient. mRNA expression levels of ZAP in tumor cell lines were retrieved from the CCLE database. **b** The protein expression levels of ZAP in HepG2, Hs578T, HeLa, and HT29 cells. **c** The infection rates of OVM-GFP in sgCtrl, sgCtrl + ZAP, ΔMXRA8 + ZAP Hs578T and HepG2 cells (MOI of 0.1) by flow cytometry (three experiments, *n* = 9; one-way ANOVA; mean ± s.d.). **d, e** The infection rates of HeLa cells transfected with siZAP or siNC and incubated with OVM-GFP virus (three experiments, *n* = 3; one-way ANOVA; mean ± s.d.). The protein expression levels of ZAP, NS3, and E1 were measured by Western blot. **f** The cell viability of HeLa tumor cells treated with OVM at the respective MOIs for 72 h was measured by MTT assay (three experiments, *n* = 9; one-way ANOVA; mean ± s.d.). *P < 0.05; **P < 0.01; ***P < 0.001

From the perspective of viral life cycle, OVM is likely to infect tumor cells with entry receptors to get in and a permissive intracellular environment to replicate. In order to test which intracellular factors reflect the oncolysis ability of OVM cooperating with the receptor MXRA8, we compared the oncolytic ability of OVM with the ZAP, ERN1 or RHOQ expression in several tumor cells using Spearman’s correlation (Fig. 5a, Supplementary Fig. 8f, g). Compared to other intracellular factors, ZAP expression could better predict the different levels of sensitivity to OVM in MXRA8 expressing tumor cells. We observed an obvious negative correlation between the oncolytic ability of OVM and ZAP expression in MXRA8-expressing tumor cells but not in MXRA8-deficient tumor cells (Fig. 5a, b). To further confirm the prediction of anti-tumor responses to OVM by the combination of MXRA8 and ZAP, ZAP was ectopically expressed on Hs578T and HepG2 cells (Supplementary Fig. 8h). The results demonstrated that the infection rate of OVM was reduced on Hs578T and HepG2 ZAP overexpressing cells, and the infectivity was further weakened on cells with both MXRA8-KO and ZAP overexpression (Fig. 5c). Consistently, knock-down of ZAP significantly promoted the infection of OVM in HeLa-MXRA8 and LoVo-MXRA8 cells, while exerted limited effect on LoVo and HeLa cells with lower MXRA8 expression (Fig. 5d, e, Supplementary Fig. 8i, j). Moreover, through ectopic MXRA8 expression and ZAP knock-down, OVM resistant tumor cells were gradually transformed into sensitive cells (Fig. 5f, Supplementary Fig. 8k).

Collectively, our data indicated that the OVM-induced oncolysis could be a comprehensive effect of MXRA8 and ZAP.

### Prediction of anti-tumor responses to oncolytic virus M1 by the combination of MXRA8 and ZAP in patient-derived tissues

**Fig. 6:**
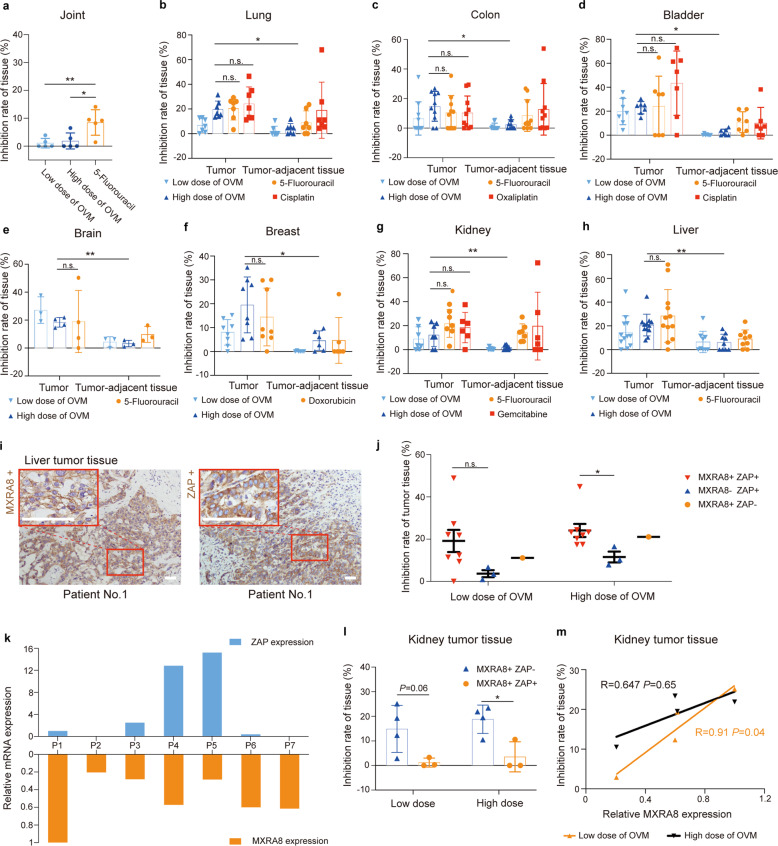
High MXRA8 expression and ZAP deficiency promote the oncolysis of OVM in patient-derived tumor tissues. **a** Inhibition rate of joint tissue treated with OVM and 5-Fluorouracil (*n* = 5). **b–h**, Inhibition rate of tumor tissue and tumor-adjacent tissue in lung (*n* = 7), colon (*n* = 8), bladder (*n* = 7), brain (*n* = 4), breast (*n* = 8), liver (*n* = 12), and kidney (*n* = 7) treated with OVM, 5-Fluorouracil, Cisplatin, Oxaliplatin, Doxorubicin, and Gemcitabine respectively. Each dot represents a single patient. Data were analyzed by one-way ANOVA. **i** Representative image cores of MXRA8 and ZAP positive immunostaining in liver tumor tissue. Image of higher magnification was shown in box. Scale bars, 50 μm. **j** Liver tumor tissue inhibition rate of groups of patients with different ZAP and MXRA8 expression (two-tailed unpaired *t*-test; mean ± s.d.). **k** Relative ZAP and MXRA8 mRNA expression of each kidney tumor tissue measured by qPCR. **l** Comparison of inhibition rate of kidney tumor tissues with high ZAP or low ZAP expression (two-tailed unpaired *t*-test; mean;± s.d.). **m** Correlation of inhibition rates and MXRA8 expression levels in kidney tumor tissue with MXRA8 expression and ZAP deficiency. *R* indicates the Pearson correlation coefficient. *P < 0.05; **P < 0.01; ***P < 0.001

In an effort to further translate OVM to the clinical use, 108 human surgical tumor or tumor-adjacent tissue specimens including joint, lung, colon, bladder, brain, breast, kidney, and liver tissues were tested for curative effect and safety evaluation of OVM. In accordance with a previous study in nonhuman primates^18^, direct OVM infection led to limited damage in joint tissues and was significantly lower than 5-Fluorouracil treatment (Fig. 6a). In tests of tumor tissues, OVM showed an overall inhibitory effect on lung, colon, bladder, brain, breast, kidney, and liver tumor tissues, and the effect of OVM was highly competitive compared with other regular chemotherapeutic agents, such as 5-Fluorouracil, Cisplatin, Oxaliplatin, Gemcitabine, and Doxorubicin (Fig. 6b–h, Supplementary Fig. 9, Supplementary Table 4). Moreover, compared to paired tumor tissues, OVM showed good tumor selectivity that caused less damage on tumor-adjacent tissues incubated with the same doses of OVM (Fig. 6b–h, Supplementary Fig. 9, Supplementary Table 4).

Although OVM showed good inhibitory effect on multiple types of tumors, we observed different outcomes of oncolysis in each tumor type at individual level (Supplementary Fig. 9). For example, the inhibition rate of liver tumor tissues ranges from 0% to 48.9% at low dose of OVM (Fig. 6h), and kidney tumor tissues from patient No. 4 and No. 5 were obviously resistant to OVM infection either in low dose or high dose, whereas 5-Flurouracil or Gemcitabine treatment still had an inhibitory effect (Supplementary Fig. 9e). In view of the combinational effect of MXRA8 and ZAP in OVM’s life cycle, we measured the expression of MXRA8 and ZAP in liver tumor tissues by IHC (Fig. 6i). The data showed that OVM had a better oncolysis on liver tumor tissues with MXRA8 positive staining compared to tumor with no MXRA8 expressing (Fig. 6j). Similarly, by using RT-qPCR, seven kidney tumor patients were divided into MXRA8^+^/ZAP^−^ (patient No. 1, 2, 6, and 7) or MXRA8^+^/ZAP^+^ (patient No. 3, 4, and 5) group (Fig. 6k). Compared to the group with high ZAP expression, tumor inhibition rate in MXRA8^+^/ZAP^−^ was significantly increased under high dose of OVM treatment (Fig. 6l). Moreover, by controlling the inhibitory impact of ZAP in kidney tumor, MXRA8 expression showed an obvious positive correlation with the sensitivity of tumor tissues to OVM treatment (Fig. 6m).

**Fig. 7:**
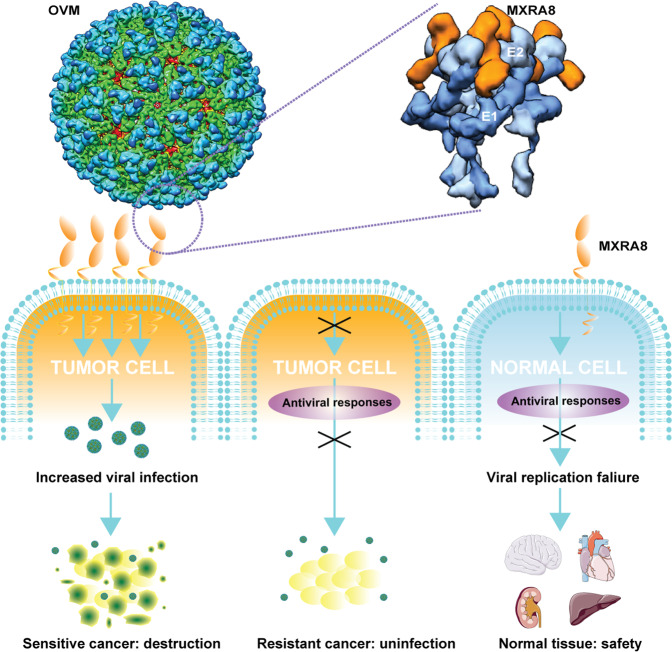
Graphical model of the tumor selectivity of OVM. OVM is a 680 Å diameter icosahedral particle with 80 trimeric spikes that assembled by 240 embedded E2–E1 heterodimers in a lipid bilayer. The E2–E1 heterodimers could directly bind MXRA8 on tumor cells, thus mediate the entry and internalization of OVM virions. OVM could effectively infect tumor cells with MXRA8 expression and a permissive intracellular environment for viral replication, which eventually lead to the lysis of tumor tissue. However, active antiviral responses, such as ZAP, and lack of MXRA8 also make some tumor cells resistant to OVM infection. In addition, the low expression of viral receptors and antiviral activities works together to build the safety of OVM in normal tissues. Above all, the tumor selectivity of OVM is mediated by the entry receptor and intracellular antiviral activity

Overall, the ex vivo culture data showed the safety and an overall inhibitory effect of OVM on several tumor types and further provided evidence for using the combination of MXRA8 and ZAP as a pair of predictive biomarkers in OVM therapy in liver and kidney tumors (Fig. 7).

## Discussion

In recent years, a growing number of OVs are moving from bench to bedside, some of which remarkably ameliorated the prognosis in patients with several notorious cancer types, making OV therapy a hopeful novel anticancer strategy^3,4^. Besides harboring the characteristics of targeted therapy and gene therapy, OV therapy is currently seen as an essential embranchment of immunotherapy by siphoning activated antitumor components in the wake of improved understanding of antitumor immunity^19^.

Like any other immunotherapies, patients respond to OV therapy differently and the overall response rate is around 20%^4,5^. The decisive translational obstacle comes from the lack of predictive biomarkers, making it hard to discriminate between responders and non-responders before initiating therapy. Although plenty of natural and genetically oncolytic viral agents have been identified or constructed^20^, few researchers have attempted to specify the biomarkers for OVs, such as deficiency of interferon-stimulated genes (ISGs) for vesicular stomatitis virus (VSV)^21^ and *K-RAS* mutation for reovirus^22^. However, since OVs are a class of unique self-amplifying biological medicine whose infection and replication highly depend on host factors^23^, a comprehensive exploration of the elusive molecular mechanisms which mediate the cancer tropism of OVs is indispensable for sculpting the biomarkers, making it still be challenging to delineate bona fide biomarkers for OVs.

In this report, we provided conclusive evidence that MXRA8 acts as the receptor and a therapeutic biomarker for OVM. MXRA8 is broadly highly expressed in multiple solid tumors, indicating that a high percentage of patients will be screened out as potential beneficiaries for OVM treatment in clinical use. This study also established a successful model for identifying the receptor of OV as a predictive biomarker by multi-cell expression profiling, cryo-EM technique, and human tumor explants. Similarly, the deficiency of ZAP, a previously identified biomarker for OVM, is also common in human cancers^9^. Moreover, the general deficiency of ZAP has promoted us to explore its previously unknown function in tumorigenesis, and we have identified ZAP as a novel tumor suppressor in colorectal cancer^24^. The widely high expression of MXRA8 in multiple solid tumors uncovered in this study might also hint a causative role in tumor progression, which may further help link predictive biomarkers of OVs to tumor pathogenesis.

Currently, most predictive biomarkers for cancer therapy that cannot self-replicate are based on one single molecule such as Herceptin for breast cancer patients with positive HER-2 expression, here we described it as “single biomarker system”. The fact that OVs depend on cellular membrane receptors to get in and intracellular factor to replicate implicates the requirement of a “dual-biomarker system” for them. The urgent need of dual biomarkers for OVs was also found in the attempt to confirm the junctional adhesion molecule-1 (JAM-1), the main cellular receptor for reovirus, as a predictive biomarker, that one of the most resistant cell lines to reovirus had the highest JAM-1 expression level^25^. In this study, we also found that only using the receptor MXRA8 or intracellular factor ZAP cannot accurately predict the efficacy of OVM in some cases, which has become the big elephant in the room during the development of precision medicine for OVM. Therefore, a “dual-biomarker system” including the expression of MXRA8 and the deficiency of ZAP is developed here, which can superiorly correlate with the efficacy of OVM in breast, liver, cervix and colon tumor cells as well as liver and kidney surgical cancer explants. This study highlights the necessity and provides an example to develop better biomarker systems for OV therapy.

The search of biomarkers for some types of cancer medicine is phenomena driven, which largely depends on the observation about the correlation between potential molecules expression or variation with the efficacy of the drugs^26^. Dissimilarly, uncovering the biomarkers for OVs needs the illustrating of key molecular mechanisms underlie the tumor selectivity of them, which renders these biomarkers as mechanism-driven ones. In this report, by focusing on the receptor recognition and intracellular replication of OVM, two essential steps in OVM’s life cycle, we established a dual therapeutic predictor system. Although this system was demonstrated by several tumor cell lines and patient-derived liver or kidney samples, more investigations on other tumor models are still needed to evaluate its potential in future clinical use. In addition, works are also needed for patient stratification on expression of MXRA8 and ZAP to precisely predict the efficacy of OVM in clinical use.

In summary, our data showed that MXRA8, the receptor for OVM, has positive impacts on the oncolytic effect of this virus. In consideration of the heterogeneity of MXRA8 and ZAP expression in cancers, we highlight the importance of using MXRA8 and ZAP as a potential dual-biomarker for predicting curative effects and selecting patients for OVM on multiple solid tumor types in clinical use.

## Materials and methods

### Cell lines and virus production

Cell lines were purchased from ATCC (HepG2, LoVo, U-87MG, and Vero cell lines), Guangzhou Cellcook Biotech (HeLa, HUVEC and BHK-21 cell lines), Guangzhou Jennio Biotech (Hs578T cell line), and Shanghai Institute of Cell Biology (769-P and HT29 cell lines). HepG2, BHK-21, and Vero cells were cultured in Eagle’s minimum essential medium (Corning) supplemented with 10% fetal bovine serum (FBS, Gibco) and 1% penicillin/streptomycin (HyClone). Hs578T cells were cultured in RPMI-1640 medium (Gibco) supplemented with 10% FBS and 1% penicillin/streptomycin. HUVEC cells were cultured in Dulbecco’s modified Eagle’s medium (Corning) supplemented with 10% FBS. All cells were maintained at 37 °C in 5% CO_2_ and tested negative for Mycoplasma infection. Oncolytic virus M1 (OVM)(M1-c6v1 strain) and OVM-GFP^27^ virus were propagated in Vero cells (OPTI-SFM, Thermo Fisher) and were provided by Guangzhou Virotech Pharmaceutical Technology Co., Ltd. The virus titer was determined by a CCID_50_ assay in BHK-21 cells.

### Statistic

R version 3.6.1 and Prism 7.0 software (GraphPad) were used for statistical analysis. Data were analyzed by unpaired Student’s *t-*test, paired Student’s *t*-test, one-way ANOVA, Tukey’s multiple comparisons test, or repeated-measures ANOVA in a general linear model, as specified in the Figure legends. *P* $<$ 0.05 was considered statistically significant. The bar charts show the mean ± standard deviation (SD).

## ^Supplementary information^

Supplementary -clean

## Data Availability

All data supporting this paper are present within the paper and/or the Supplementary Materials. The obtained cryo-EM density maps were deposited into the Electron Microscopy Data Bank (EMDB) under the following accession numbers: OVM, EMD-30509; OVM-MXRA8 complex and diff map, EMD-30510.
