# Supplementary material for: Identification of the receptor of oncolytic virus M1 as a therapeutic predictor for multiple solid tumors
Source: Signal Transduct Target Ther. 2022 Apr 8;7:100. doi: 10.1038/s41392-022-00921-3 (PMC8989880; doi:10.1038/s41392-022-00921-3)
Supplement: Supplementary file 1 — Supplementary -clean [file 41392_2022_921_MOESM1_ESM.docx]

Supplementary Materials for

Identification of the receptor of oncolytic virus M1 as a therapeutic predictor for multiple solid tumors

Deli Song^1^, Xudong Jia^2^, Xincheng Liu^1^, Linyi Hu^1^, Kaiying Lin^1^, Tong Xiao^1^, Yangyang Qiao^1^, Jiayu Zhang^1^, Jia Dan^1^, Chunwa Wong^1^, Cheng Hu^3^, Ke Sai^4^, Shoufang Gong^5^, Max Sander^5^, Runling Shen^1^, Xiaoyu chen^1^, Xiaoting Xiao^1^, Jiehong Chen^1^, Yanming Zhang^1^, Cailv Wei^1^, Xiao Xiao^1^, JianKai Liang^1^, Qinfen Zhang^2^, Jun Hu^6^, Wenbo Zhu^1^, Guangmei Yan^1^, Yuan Lin^1^, Jing Cai^1^

Correspondence to: [liny96@mail.sysu.edu.cn](mailto:liny96@mail.sysu.edu.cn) and [caij53@mail.sysu.edu.cn](mailto:caij53@mail.sysu.edu.cn).

**This PDF file includes:**

Materials and Methods

Figures. S1 to S9

Tables S1 to S4

Materials and Methods

**CRISPR-Cas9-mediated MXRA8 gene knockout**

MXRA8 knockout cell lines were generated by using CRISPR-Cas9 gene editing technology. The two single-guide RNA (sgRNA) sequences targeting MXRA8 were sgRNA-1 (5'-GGCGCGGATGCCTTTGAGCG-3') and sgRNA-2 (5'-GTCCGCCTGGAGGTCACCGA-3') ^1^. The sgRNAs were cloned into a Cas9 expression plasmid (GeneCopoeia, pCRISPR-LvSG06) and packaged into lentiviral particles. Hs578T and HepG2 cells infected with lentivirus were screened with puromycin for 7 days and isolated by limiting dilution.

**Plasmids and ectopic expression experiment**

For the MXRA8 and ZAP overexpression assay, human MXRA8 transcript (NM_32348) or ZAP transcript (NM_020119) with a Flag tag at the C-terminus was synthesized and integrated into the pEZ-Lv216 vector (GeneCopoeia) and packaged with a lentiviral expression system (GeneCopoeia). In order to avoid re-cutting by the previous sgRNA, mutated MXRA8 transcripts (5'-GTCCGCCTGGAGGTCACCGAC-3' to 5'-GTAAGGCTAGAAGTGACAGAT-3', 5'-GGCGCGGATGCCTTTGAGCGC-3' to 5'-GGAGCAGACGCGTTCGAACGA-3') were cloned into the pReceiver-Lv158 vector (GeneCopoeia) for the trans-complementation of MXRA8 in MXRA8 knockout cell lines. Cells infected with lentivirus were screened with puromycin for 7 days. The ectopic expression was validated by Western blotting.

**Cell viability assay**

In brief, 4×10^4^ cells were seeded in 48-well plates and cultured overnight. After treatment, the medium was replaced with medium containing 3-(4,5-dimethylthiazol-2-yl)-2,5-diphenyltetrazolium bromide (MTT, 0.5 mg/ml, Sigma) and incubated at 37°C for another 3 h. The old medium was discarded, and the MTT precipitate was dissolved in 200 μl of DMSO. The optical absorbance was measured at 490 nm using a microplate reader (BioTek, Synergy H1).

**Weighted gene co-expression network analysis**

The gene co-expression network analysis was conducted using WGCNA (version 3.6.3) to identify co-expressed genes in 14 breast tumor cell lines. Transcriptome data of breast tumor cell lines were obtained from Broad Institute Cancer Cell Line Encyclopedia (CCLE), and only the most-varying genes (3718) in 14 breast tumor cell lines were used for module construction. In order to ensure the network satisfied a scale-free topology, a soft power value of 9 was chosen from index by using the pichSoft Threshold function. Co-expression modules were identified using blockwise Modules function with the following settings: soft threshold power β=9, deep Split=0, minimum module size=20, merge cut height =0.25. For each Module’s eigengene, a Spearman’s correlation analysis was used to identify modules significantly correlated to cell viability under OVM treatment. After the correlation analysis, leading genes in the module that significantly associated with the oncolytic effect were further evaluated by KEGG analysis.

**Western blotting**

After washing with PBS once, cells were lysed in 200 μl of M-PER Mammalian Protein Extraction Reagent (Thermo Fisher) with protease inhibitors (Targetmol) on ice followed by centrifugation at 12,000 × g for 10 min. Then, the protein concentration in the supernatant was measured using a BCA kit (Thermo Fisher), and the supernatant was diluted in SDS-PAGE sample loading buffer (Beyotime). Samples were heated at 100°C for 5 min, separated by SDS-PAGE and transferred to PVDF membranes by using an eBlot L1 system (GenScript). Membranes were blocked with 5% nonfat milk for 1 h at room temperature. Immunoblotting was performed using antibodies specific for Flag (Sigma,1:200), PERK (CST, 1:1000), pPERK (CST, 1:1000), EIF2A(CST, 1:1000), pEIF2A (CST, 1:1000), ZAP (Thermo, 1:1000), GAPDH (arigo, 1:5000), α-Tubulin (arigo, 1:5000), E1 (1:1000), NS3 (1:1000), β-actin (arigo, 1:5000) and appropriate HRP-conjugated secondary antibodies (arigo). Immunoreactions on the membranes were visualized by using Immobilon Western Chemiluminescent HRP Substrate in a ChemiDoc XRS+ System (Bio-Rad). Antibodies for OVM’s E1 and NS3 proteins were produced as described previously ^2^.

**MXRA8-His extracellular protein production and anti-MXRA8 antibody development**

For the production of MXRA8-His protein, a cDNA fragment encoding the extracellular domain of human MXRA8 (AA residues 20-310, NM_001282585) with a His-tag at the C-terminus was synthesized (Sino Biological), inserted into the pET-21a vector (Sino Biological) and sequenced. The purified plasmid was then transfected into BL21 (Takara) cells at 37°C and 250 rpm. The supernatant of transfected cells was collected and purified by Ni-NTA column (20 ml) affinity chromatography (Qiagen). The purity of MXRA8-His was further validated through SDS-PAGE.

For the development of the anti-MXRA8 antibody, 50 µg of purified MXRA8 in equal volumes of incomplete Freund’s adjuvant (BD) was injected into BALB/c mice in the abdominal region. After three vaccinations, the serum of mice was sampled for antibody titer measurement through Western blot analysis. Hybridoma cells were obtained by fusing mouse spleen cells with mouse myeloma cells (SP20) via electrofusion (BTX). Supernatants of hybridoma cells were collected and purified by Ni-NTA column (20 ml) affinity chromatography. To evaluate antibody specificity, supernatants were serially diluted and tested with several MXRA8-positive (HepG2, HeLa cells with MXRA8 overexpression) and MXRA8-negative (HeLa control cells) samples by Western blotting. Clones with good performance in the assay were selected for further study.

**RT-qPCR**

RNA of virions or cells was extracted with TRIzol reagent (Ambion) and reverse transcribed into cDNA with oligo(dT) and RevertAid Reverse Transcriptase (Thermo Fisher). Quantitative PCR was conducted with SuperReal PreMix SYBR Green (Tiangen) using β-actin as the internal control. The sequences of the primers used for qPCR were as follows:

β-actin: sense, 5’-GATCATTGCTCCTCCTGAGC-3’ and antisense, 5’-ACTCCTGCTTGCTGATCCAC-3’;

OVM NS1: sense, 5’-GTTCCAACAGGCGTCACCATC-3’ and antisense, 5’-ACACATTCTTGTCTAGCACAGTCC-3’;

MXRA8: sense, 5’- TCTCTGCCCCATCCCTACCCT-3’ and antisense, 5’-ACCCCAAACCAACTTCACTCCCT-3’.

ZAP: sense, 5’- TCACGAACTCTCTGGACTGAA-3’ and antisense, 5’-ACTTTTGCATATCTCGGGCATAA-3’.

**RNA interference**

Specific siRNAs purchased from Ribobio (Guangzhou, China) were transfected using Lipofectamine RNAiMAX (Thermo Fisher) according to the manufacturer’s instructions. Cell medium was replaced with Opti-MEM (Thermo Fisher).

**OVM binding and internalization assay**

Before the assay, 5×10^5^ cells were seeded in 6-well plates overnight. To verify OVM binding, virions (MOI of 20) were incubated with cells for 1 h at 4°C. Cells were washed five times to remove unbound virions, after which RNA was extracted with TRIzol reagent (Ambion) followed by quantitative PCR. For the internalization assay, washed cells were suspended in medium containing 15 mM NH_4_Cl and moved to incubators at 37°C for 1 h. Cells were treated with trypsin-EDTA and proteinase K at 4°C for 1 h. After 3 cycles of centrifugation and washing, RNA was extracted and quantified as described above.

**Blocking assay**

For the purified human MXRA8-His extracellular domain protein blocking assay, OVM (MOI of 4) was incubated with serially diluted MXRA8 protein for 3 h at 4°C. PD-L1 protein (Sino Biological) at indicated concentration was used as negative control. The mixture was added to 96-well plates seeded with HeLa+MXRA8 or Hs578T cells, and the percentages of M1-GFP-infected HeLa+MXRA8 and Hs578T cells were determined by flow cytometry at 24 h (HeLa+MXRA8) or 12 h (Hs578T).

**Immunohistochemistry assay**

Tumor sections were dewaxed with xylene, hydrated with ethanol of descending concentration, immersed in H_2_O_2_-methanol and washed with phosphate-buffered saline. After washing, tumor sections were probed with antibody specific for ZAP (Sigma, 1:200) or MXRA8 (Sigma, 1:200) and incubated overnight. Then, the tumor sections were washed and incubated with mouse anti- rabbit IgG for 2 hours at room temperature. With streptavidin/peroxidase complex and diaminobenzidine, immunostainings were visualized and further counterstained with haematoxylin. The results of immunohistochemistry assay were further analyzed and verified by pathologists.

**Genomic viral RNA transfection**

To assess OVM RNA replication in cells with or without MXRA8 expression, capped genomic M1-GFP RNA generated with the RiboMAX^TM^ Large Scale RNA Production System-SP6 (Promega) was transfected into control, MXRA8-overexpressing or ΔMXRA8 cells. Cells were transfected with purified RNA by using Lipofectamine MessengerMax reagent (Thermo Fisher). The medium was supplemented with 15 mM NH_4_Cl to prevent subsequent rounds of viral infection. The transfection rates were measured by determining the expression level of GFP using flow cytometry.

**Cryo-EM sample preparation, data collection and processing**

To generate the OVM-MXRA8 complex, an excess of MXRA8-His extracellular domain proteins were incubated with purified OVM at 4°C for more than 6 hours. A 3 μl liquot of purified OVM virion, and OVM-MXRA8 complex were applied to freshly glow-discharged carbon-coated Quantifoil R1.2/1.3 copper grids (Quantifoil Inc.) respectively, followed by rapid flash freezing in liquid ethane using a Vitrobot Mark IV (FEI).

Cryo-EM datasets were collected at 200 kV with a Tecnai F20 (TF20) electron microscope (EM) (FEI) equipped with an Eagle charge-coupled device (CCD) camera (FEI). Micrographs were acquired manually with defocus values ranging from 1 to 3 µm at a nominal magnification of 62,000×, resulting in a final pixel size of 1.78 Å.

Both OVM and OVM-MXRA8 complex data were processed with the same method. Contrast transfer function (CTF) parameters were estimated using *Gctf ^3^.*Raw particles were picked from micrographs using the e2boxer.py program in the *EMAN2* package and were subjected to reference-free 2D classification using *relion-3.0^4^*. Good classes were selected for initial model building by *e2initialmodel.py* in *EMAN2*. The initial models were further refined using *relion-3.0*, yielding the final reconstructions. The resolution of the reconstructions was estimated using the “gold-standard” ^5, 6^. Fourier shell correlation (FSC), based on the 0.143 criterion ^7^. Structures were visualized and compared using UCSF*Chimera ^8^*.

We docked the atomic model of the MXRA8 ectodomain (PBD: 6JO8) into the additional densities in map OVM-MXRA8 complex using UCSF*Chimera* “Fit in map” tools. The cross correlation values between the additional densities and the MXRA8 density simulated from the PDB model with the same resolution were used for identification of the ectodomain’s orientation.

**Animal models**

Mouse studies were evaluated and approved by the Animal Ethical and Welfare Committee of Zhongshan School of Medicine, Sun Yat-sen University. Four-week-old female BALB/c-nu/nu mice (GemPharmatech) were subcutaneously implanted in the right flank with 1.5×10^6^ HeLa vector cells, 1.5×10^6^ HeLa MXRA8 cells, 5×10^6^ HepG2 ΔMXRA8 cells, 5×10^6^ HepG2 control cells, 2×10^6^ HT29 vector cells or 2×10^6^ HT29 MXRA8 cells. After 1-2 weeks, mice with appropriately sized tumors (50-150 mm^3^) were selected and randomized into designed groups. Mice were then intravenously injected with sterile PBS and OVM (1×10^7^ pfu/ml) in a total volume of 300 µl once daily for 10 days. The tumor volume was calculated according to the following formula:

$$Tumor volume (\mathrm{mm}^{3})=\frac{Tumor length\times{Tumor width}^{2}}{2}$$

Tumor measurements were performed by researchers blinded to the group allocations. Mice were euthanized by pentobarbital injection when the tumor reached the endpoint size (more than 1000 mm^3^), and the survival rate reflected this endpoint.

**Human tumor MXRA8 mRNA analysis**

RNA sequencing data showing the expression levels of MXRA8 in human tumor tissues and matched non-tumor tissues were obtained from The Cancer Genome Atlas (TCGA) and Genotype Tissue Expression Project (GTEX) and were downloaded from UCSC Xena (https://xenabrowser.net/) and shown as log_2_ (TPM+1) values. For tumor types lacking corresponding healthy tissues ($\leq$ 9 individuals) in the TCGA project, non-tumor tissues from TCGA and GTEX were combined for analysis. For tumor types containing enough non-tumor tissue samples ($\geq$ 10 individuals), tumor tissues were compared with matched non-tumor tissues from TCGA. The number of cases used for analysis is specified in the Figure legend.

**RNA-scope assay**

Expression of MXRA8 was detected by a probe targeting MXRA8 mRNA. Briefly, tumor tissue sections of 4 μm thickness were dewaxed in xylene and then dehydrated in descending concentrations of ethanol. Samples were immersed in H_2_O_2_ at room temperature for 10 min and washed 3 times in RNase-free H_2_O. Tissue sections were then incubated in RNA-scope Target Retrieval Reagents (Advanced Cell Diagnostics) at 98°C for 15 min, washed 3 times with RNase-free H_2_O and then treated with protease (Advanced Cell Diagnostics) at 40°C for 30 min in a HybEZ hybridization oven. After the protease was removed, target probes were hybridized with the sample for 2.5 h; AMP1-AMP6 were hybridized with tissue slides according to the manufacturer’s instructions (<https://www.acdbio.com/support.)> in the HybEZ hybridization oven. After each hybridization step, the slides were washed with wash buffer (Advanced Cell Diagnostics) for three times. Then, the slides were hybridized with hematoxylin and incubated with PBS for 1 min. Finally, the slides were immersed in 95% ethyl alcohol for 2 min and sealed with resin. Each sample was analyzed in parallel with positive probes and negative probes to ensure the quality of the results. Tissue sections were examined under a standard bright field microscope at 20-40× magnification

**Ex vivo experiments**

The ex vivo anticancer activity of OVM was evaluated through tissue culture-end point staining-computer image analysis (TECIA) ^9^. All patient-derived tumor tissues were sampled from consenting patients who underwent tumor resection. Tissue collection was performed upon receipt of informed consent from patients. This study was approved by the ethics review committee of Sun Yat-sen University (Guangzhou, China), The Third Affiliated Hospital of Sun Yat-sen University (Guangzhou, China) and Sun Yat-sen University Cancer Center (Guangzhou, China). Tissue samples were divided manually into blocks of approximately 1 mm^3^ and placed into single wells of 24-well plates containing 1.5 ml of DMEM supplemented with 15% FBS at 37°C in 5% CO_2_. After 24 hours, samples were treated with a high dose of OVM (4×10^7^ pfu/well), a low dose of OVM (2×10^6^ pfu/well), 5-Fluorouracil (120 μg/ml), Cisplatin (5 μg/ml), Oxaliplatin (10 μg/ml), Gemcitabine (30 μg/ml), Doxorubicin (5 nM) or OPTI-SFM (negative control) for 72 hours. Before adding MTT reagent, the tissues were measured in an image analysis system, and the mean area of transmitted illumination in the image was recorded as the A-score. Then, 100 μl of MTT (5 mg/ml) was added to each well and cultured for 4 hours at 37°C in 5% CO_2_. The blue area was evaluated by the staining intensity and measured using the image analysis system, and the mean blue area for each treatment was recorded as the BA score. The therapeutic efficacy was calculated as the percentage inhibition using the following formula:

$$\mathrm{Inbibition}\left( \% \right)=\left( 1-\frac{BA-treated sample /A- treated sample}{BA- control sample/A- control sample} \right)\times100\%$$

Every treatment of each sample was tested in quadruplicate.


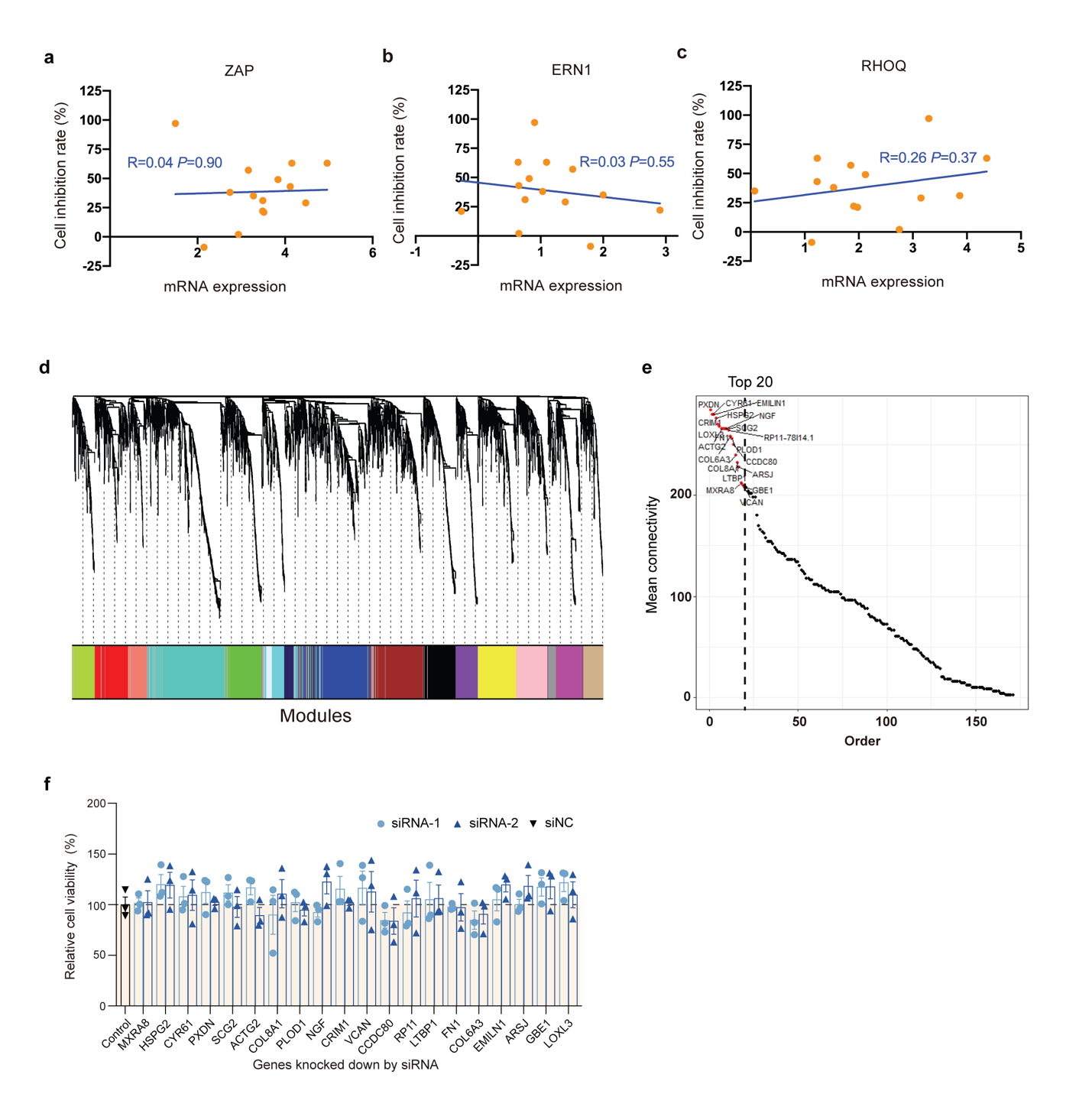


**Figure S1. A cluster of genes contributes to the oncolysis of OVM on breast tumor cells.** **a - c** Correlation of inhibition rate of OVM in 14 breast tumor cell lines and ZAP (a), ERN1 (b) or RHOQ (c) expression levels by Pearson correlation coefficient. **d** Co-expression networks were generated, and highly correlated genes within one branch were clustered into a module and assigned a color. **e** The rank of genes in turquoise co-expression module. Each dot represents a single gene. The y axis shows the mean connectivity of gene in turquoise module, and x axis represents the order of genes based on connectivity value. **f** Hs578T cells were transfected with siRNAs targeting to the top 20 differentially expressed genes and analyzed for cell viability by MTT (3 experiments, n = 3; one-way ANOVA; mean ± s.d.). * *P* < 0.05; ** *P* < 0.01; *** *P* < 0.001.


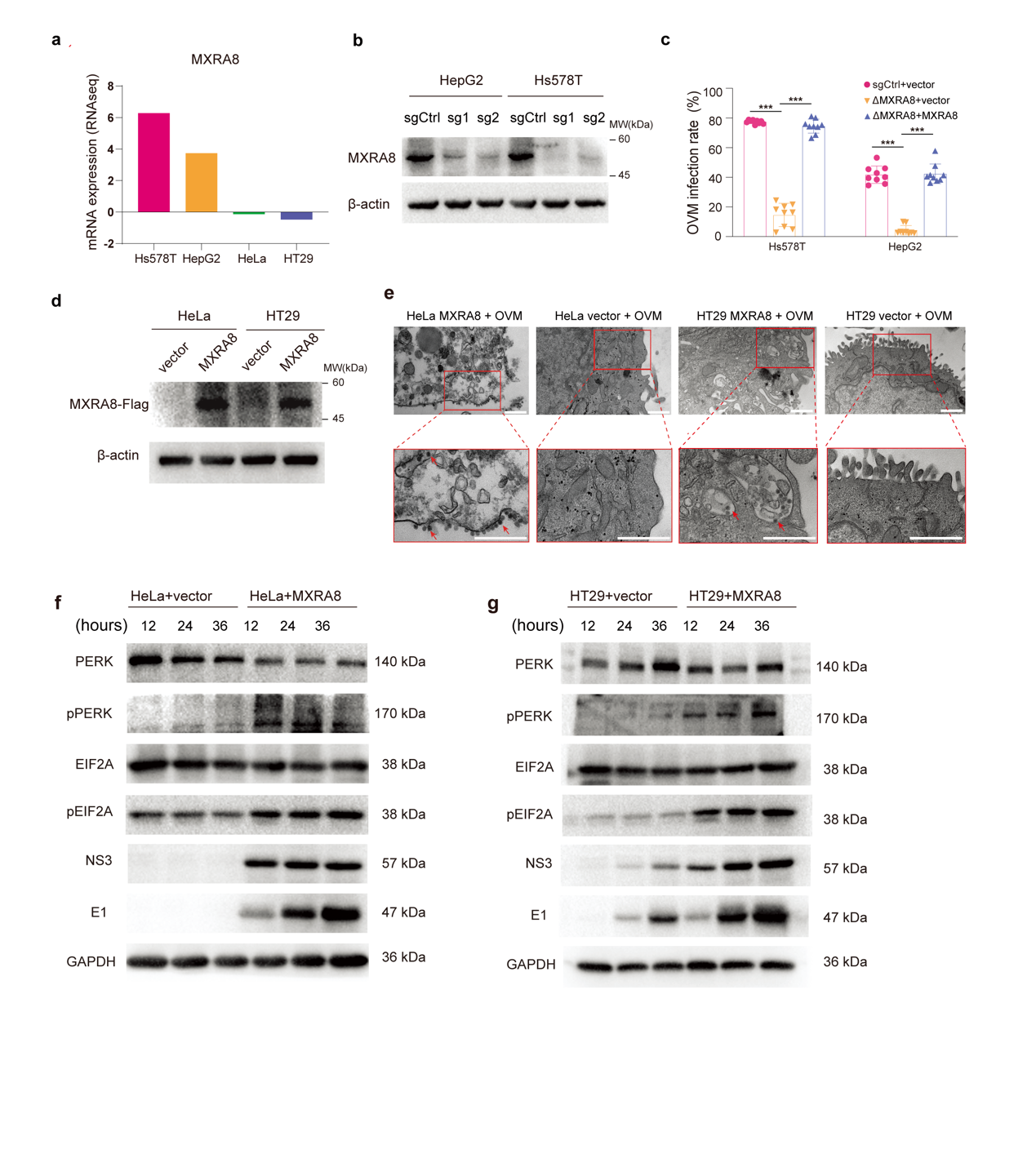


**Figure S2. MXRA8 contributes to the oncolysis of OVM on tumor cells.** **a** The mRNA level of MXRA8 in four tumor cell lines from the CCLE database. **b** The protein level of MXRA8 in HepG2 and Hs578T cells with MXRA8 gene knockout by the CRISPR-Cas9 system. **c** The infection rates of M1-GFP in control, ΔMXRA8 (sgRNA-2) and ΔMXRA8 (sgRNA-2)+MXRA8 Hs578T or HepG2 cells (MOI of 0.1 for 48 h at 37°C) by flow cytometry (3 experiments, n = 9; one-way ANOVA; mean ± s.d.). **d** The protein level of MXRA8-Flag in HeLa, HeLa+MXRA8, HT29 and HT29+MXRA8 cells. **e** HeLa+MXRA8, HeLa+vector, HT29+MXRA8 and HT29+vector cells were treated with OVM at a MOI of 3 for 48 hours; samples were prepared by conventional ultrathin sectioning methods and observed with a transmission electron microscope. The red arrows indicated M1 progeny virions. Scale bars, 1 μm. **f** HeLa+MXRA8 and HeLa+vector cells were infected with OVM (MOI=1). **g** HT29+MXRA8 and HT29+vector cells were infected with OVM (MOI=1). Proteins in (f) and (g) were examined by using Western blot. *P* < 0.05; ** *P* < 0.01; *** *P* < 0.001.

**
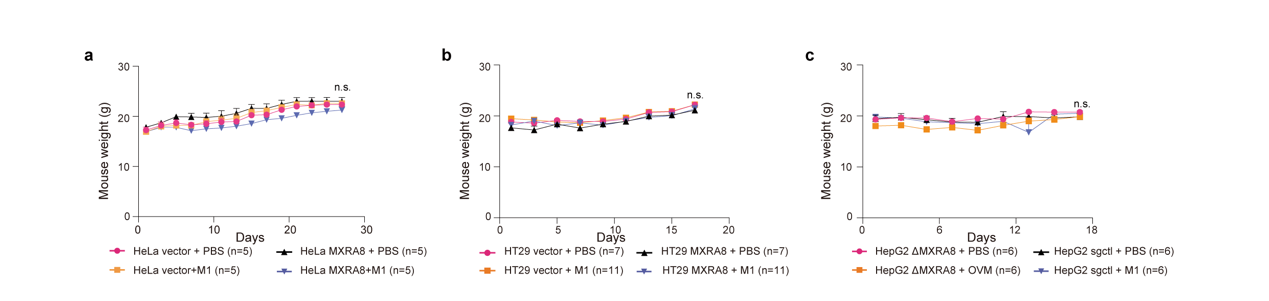
**

**Figure S3. Body weight of tumor bearing mice. a-c** body weight of mouse subcutaneously implanted with HeLa, HT-29 and HepG2 cells.


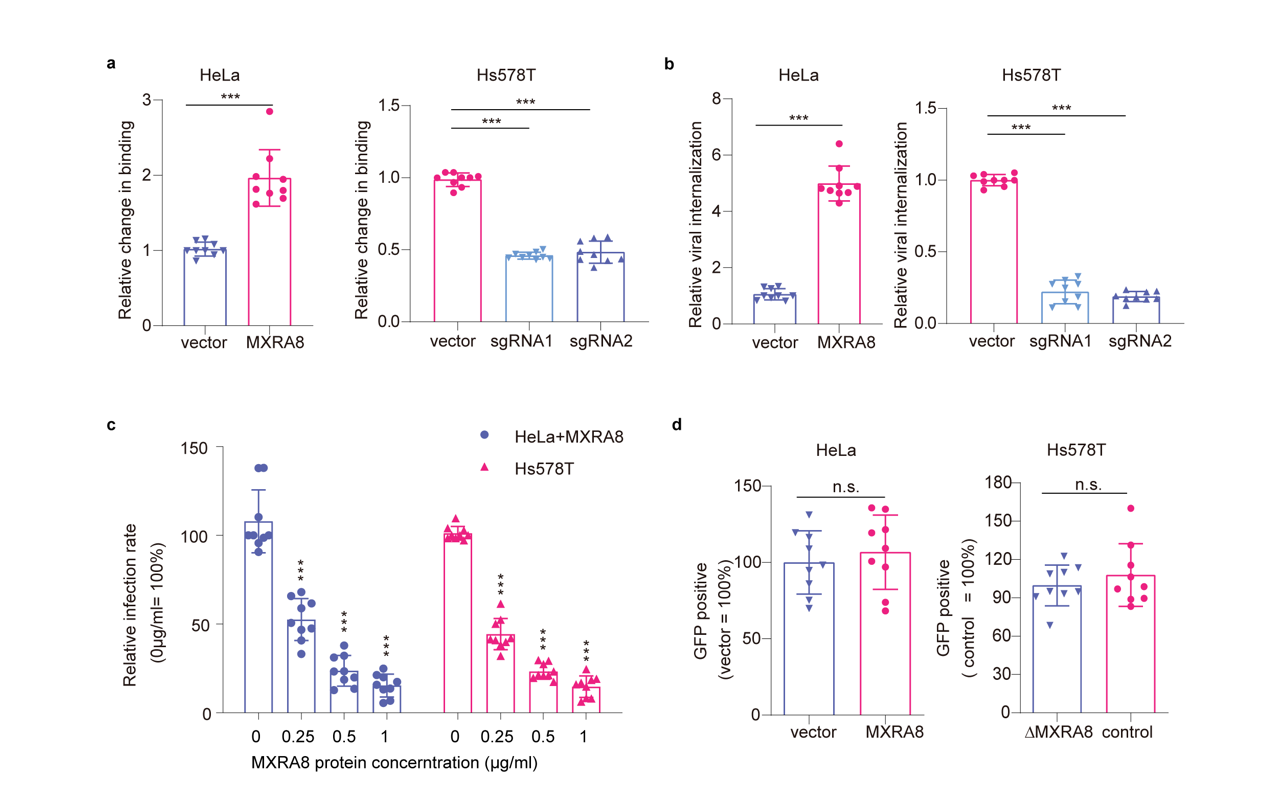


**Figure S4. MXRA8 acts a key role in the entry pathway of OVM infection. a, b** The binding and internalization ability of OVM in tumor cells with or without MXRA8 expression were assessed by measuring the amount of residual OVM RNA in treated cells using RT-PCR (3 experiments, n = 9; one-way ANOVA; mean ± s.d.). **c** M1-GFP virus was incubated with the MXRA8 extracellular protein at various concentrations before infection of Hs578T cells and HeLa+MXRA8 cells. Relative infection rate was measured by flow cytometry (3 experiments, n = 9; one-way ANOVA; mean ± s.d.). **d** RNA of M1-GFP virus was directly transfected into HeLa and Hs578T cells with or without MXRA8 expression. Replication of M1-GFP virus was detected by GFP expression using flow cytometry (3 experiments, n = 9; one-way ANOVA; mean ± s.d.). n.s., Nonsignificant. * *P* < 0.05; ** *P* < 0.01; *** *P* < 0.001.


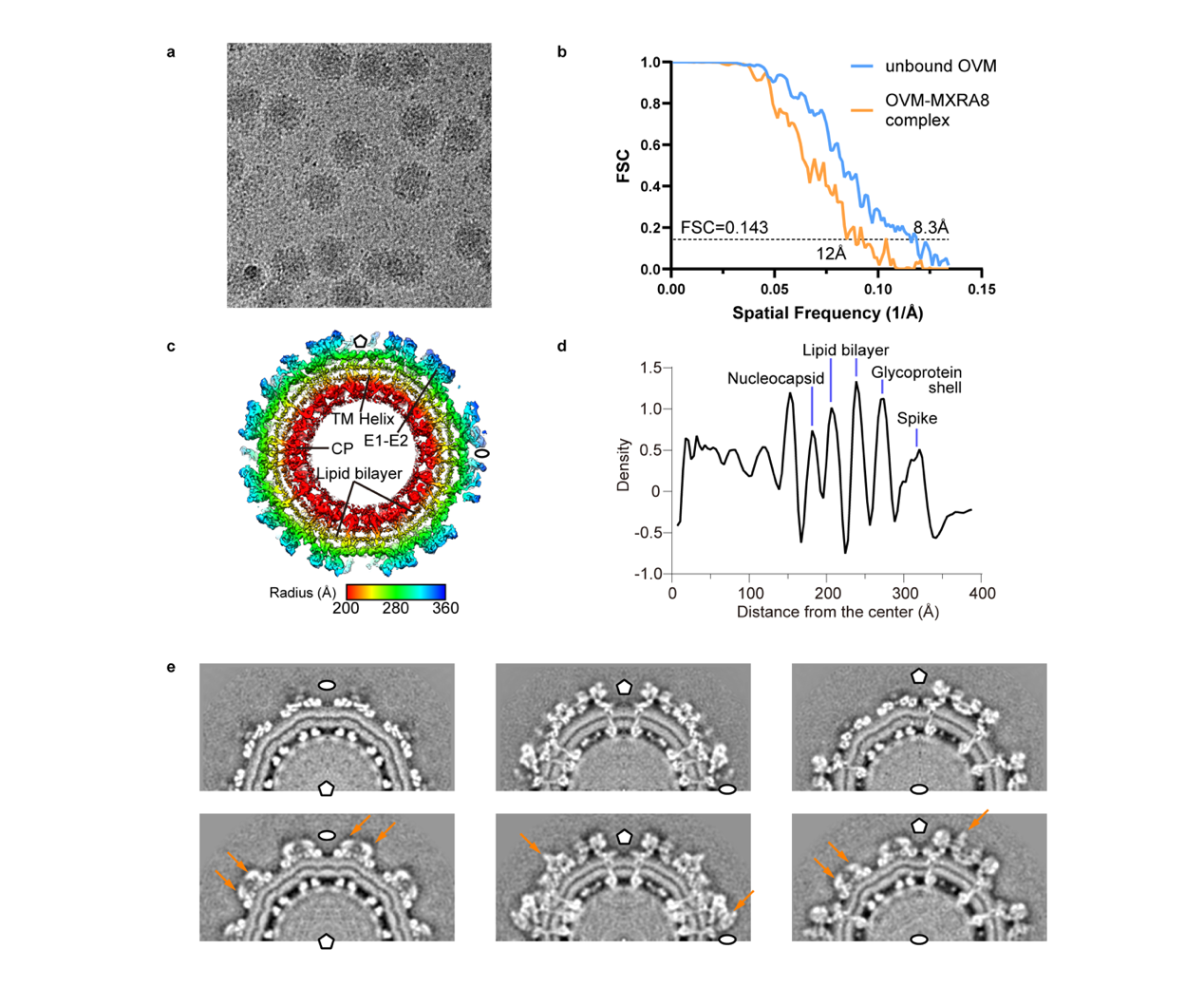


**Figure S5. The structures of OVM and OVM-MXRA8 complex by cryo-EM. a** A typical charge-coupled device (CCD) micrograph of OVM embedded in vitreous ice. **b** The gold standard Fourier shell correlation (FSC) curves of the final cryo-EM maps. The unbound OVM and the OVM-MXRA8 complex are indicated in blue and orange, respectively. **c** The central cross section through the cryo-EM map of OVM. The major structural elements are labeled. The map is radially colored. CP, capsid. TM Helix, transmembrane helix. **d** One-dimensional radial density profile of OVM. The peaks of the major elements are indicated by blue lines. **e** The central cross sections through the cryo-EM map of OVM (top) and the OVM-MXRA8 complex (bottom). The densities of MXRA8 are indicated by orange arrows. The locations of the icosahedral five- and two-fold axes are marked with pentagons and ellipses, respectively, in **c** and **e**.


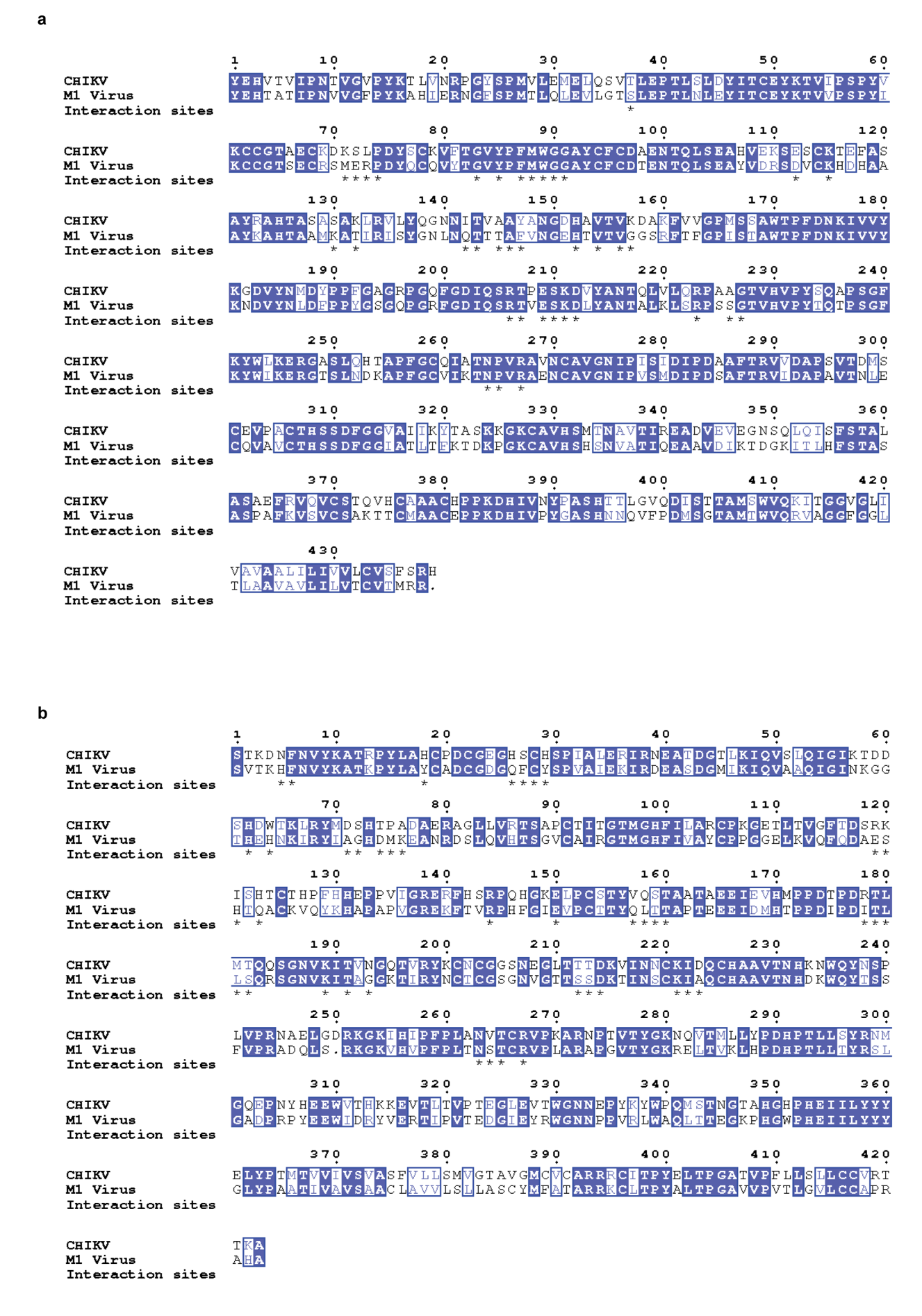


**Figure S6. Amino acid sequence alignment of the glycoproteins of CHIKV (ABX40011) and OVM.** **a** Amino acid sequence alignment of the E1 glycoproteins. **b** Amino acid sequence alignment of the E2 glycoproteins. The figure was generated using ESPript 3.0 ^10^. “Interaction sites” refer to the interaction positions of CHIKV and the MXRA8 protein. Blue boxes, 100% conserved; blue outlined boxes and blue letters, homologous residues; black letters, non-conserved residues.


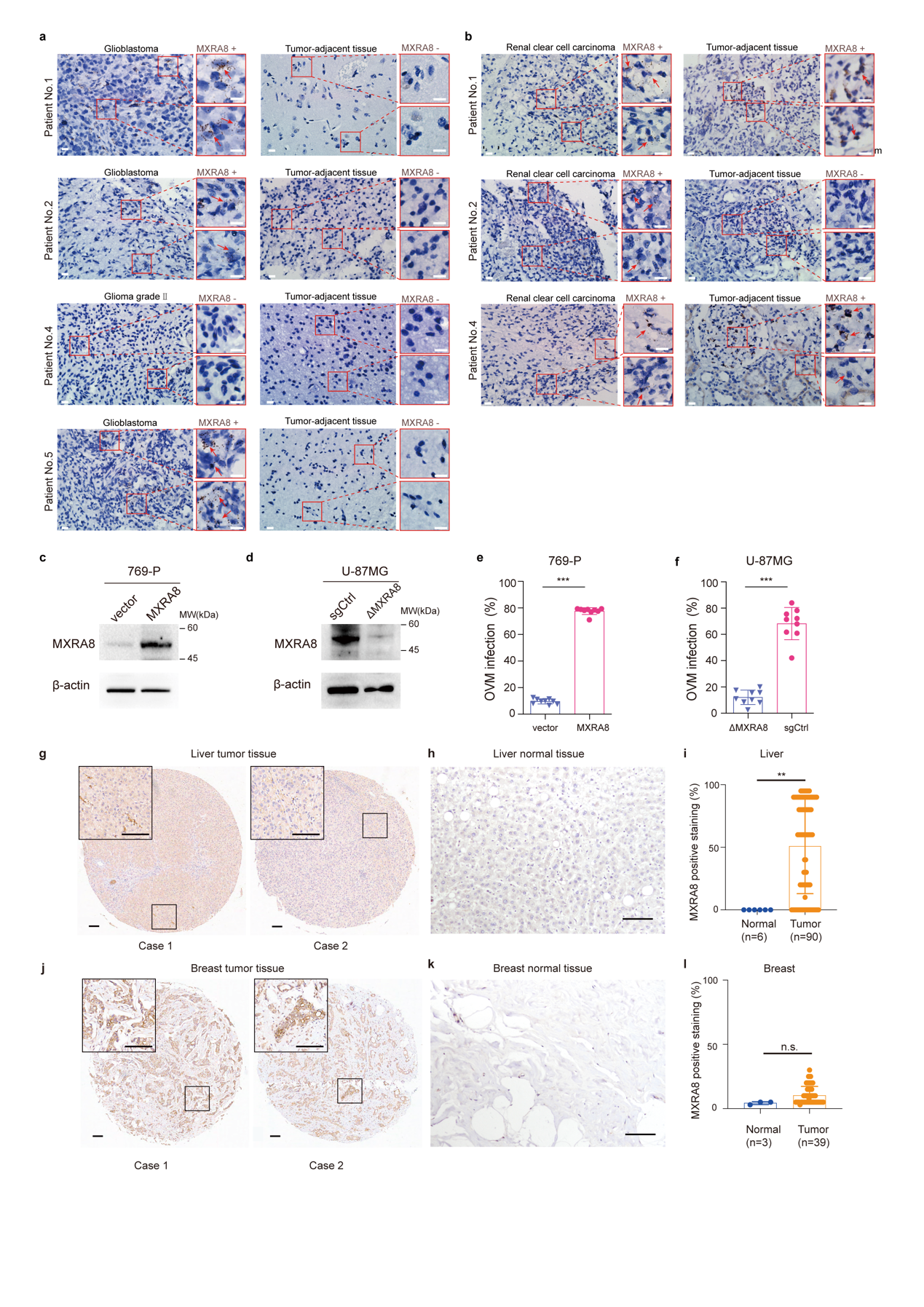


**Figure S7. MXRA8 expression in patient-derived tumor samples detected by RNA-scope and IHC. a** Glioma tissues and tumor-adjacent tissues were stained with a chromogen (DAB) and reacted with probes against MXRA8 (n=4). **b** MXRA8 in renal clear cell carcinoma tissues and tumor-adjacent tissue was detected as described in **a** (n=3). The red arrows point to the positive signal of MXRA8 in the respective images. According to the instructions, the brown spots in the images are considered MXRA8-positive signals. Scale bars, 20 μm. To control the quality of the RNA-scope results, all samples were treated in parallel with positive and negative controls. **c, d** The protein level of MXRA8 in 769-P MXRA8-overexpressing and U-87MG MXRA8-knock out cells. **e** The infection rates of M1-GFP in control and 769-P MXRA8-overexpressing cells (MOI of 1 for 24 h at 37°C) (3 experiments, n = 9; one-way ANOVA; mean ± s.d.). **f** The infection rates of M1-GFP in control and ΔMXRA8 (sgRNA-1) U-87MG cells (MOI of 0.1 for 48 h at 37°C) by flow cytometry (3 experiments, n = 9; one-way ANOVA; mean ± s.d.). **g** Representative cores of MXRA8 positive immunostaining in liver tumor tissue. Scale bars, 100 μm. **h** Liver normal tissues were evaluated through immunohistochemistry for MXRA8. Scale bars, 100 μm. **i** Statistic analysis of MXRA8 positive staining in liver tumor and normal tissue (two-tailed unpaired *t*-test; mean ± s.d.). **j** Representative cores of MXRA8 positive immunostaining in breast tumor tissue. Scale bars, 100 μm. **k** breast normal tissues were evaluated through immunohistochemistry for MXRA8. Scale bars, 100 μm. **l** Statistic analysis of MXRA8 positive staining in breast tumor and normal tissue (two-tailed unpaired *t*-test; mean ± s.d.). n.s., Nonsignificant. * *P* < 0.05; ** *P* < 0.01; *** *P* < 0.001.


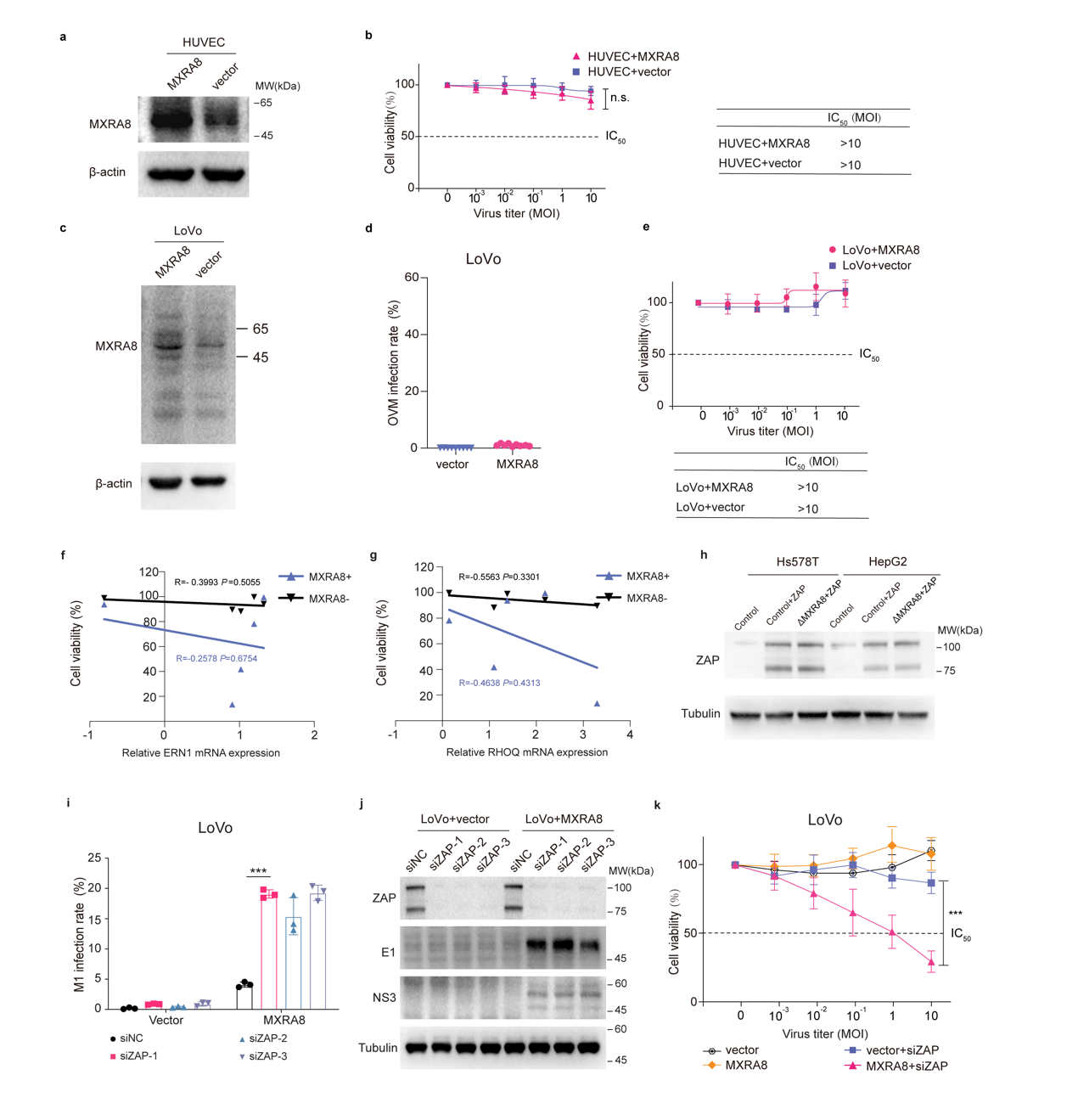


**Figure S8. The tumor selectivity of OVM depends on a combinational effect of MXRA8 and ZAP. a** The protein expression levels of MXRA8 in HUVEC vector and HUVEC MXRA8 cells. **b** HUVEC vector and HUVEC MXRA8 cells were treated with OVM at the respective MOIs for 72 h, cell viability was evaluated by MTT assay (3 experiments, n = 9; one-way ANOVA; mean ± s.d.). **c** The protein expression levels of MXRA8 in LoVo vector and LoVo MXRA8 cells. **d** LoVo vector and LoVo MXRA8 cells were treated with 1 MOI OVM for 36 h, after which infection rates were measured by flow cytometry (3 experiments, n = 9; one-way ANOVA; mean ± s.d.). **e** LoVo vector and LoVo MXRA8 cells were treated with OVM at the respective MOIs for 72h, cell viability was evaluated by MTT assay (3 experiments, n = 9; one-way ANOVA; mean ± s.d.). **f, g** Spearman’s correlation coefficient of cell viability under OVM treatment and ERN1(f) or RHOQ (g) expression levels in Hs578T, HepG2, HT29 and HeLa cell lines. **h** The protein expression levels of ZAP in sgCtrl, sgCtrl+ZAP, ΔMXRA8+ZAP Hs578T and HepG2 cells. **i, j** LoVo cells were transfected with siNC or siZAP and infected with OVM at 10 MOI for 30h. The infection rate was detected by flow cytometry (3 experiments, n = 3; one-way ANOVA; mean ± s.d.). The protein expression of ZAP, E1 and NS3 were measured by Western blot. **k** The viability of tumor cells treated with siRNA and OVM (72 h) was evaluated by MTT assay (3 experiments, n = 9; one-way ANOVA; mean ± s.d.). n.s., Nonsignificant; * *P* < 0.05; ** *P* < 0.01; *** *P* < 0.001.

**
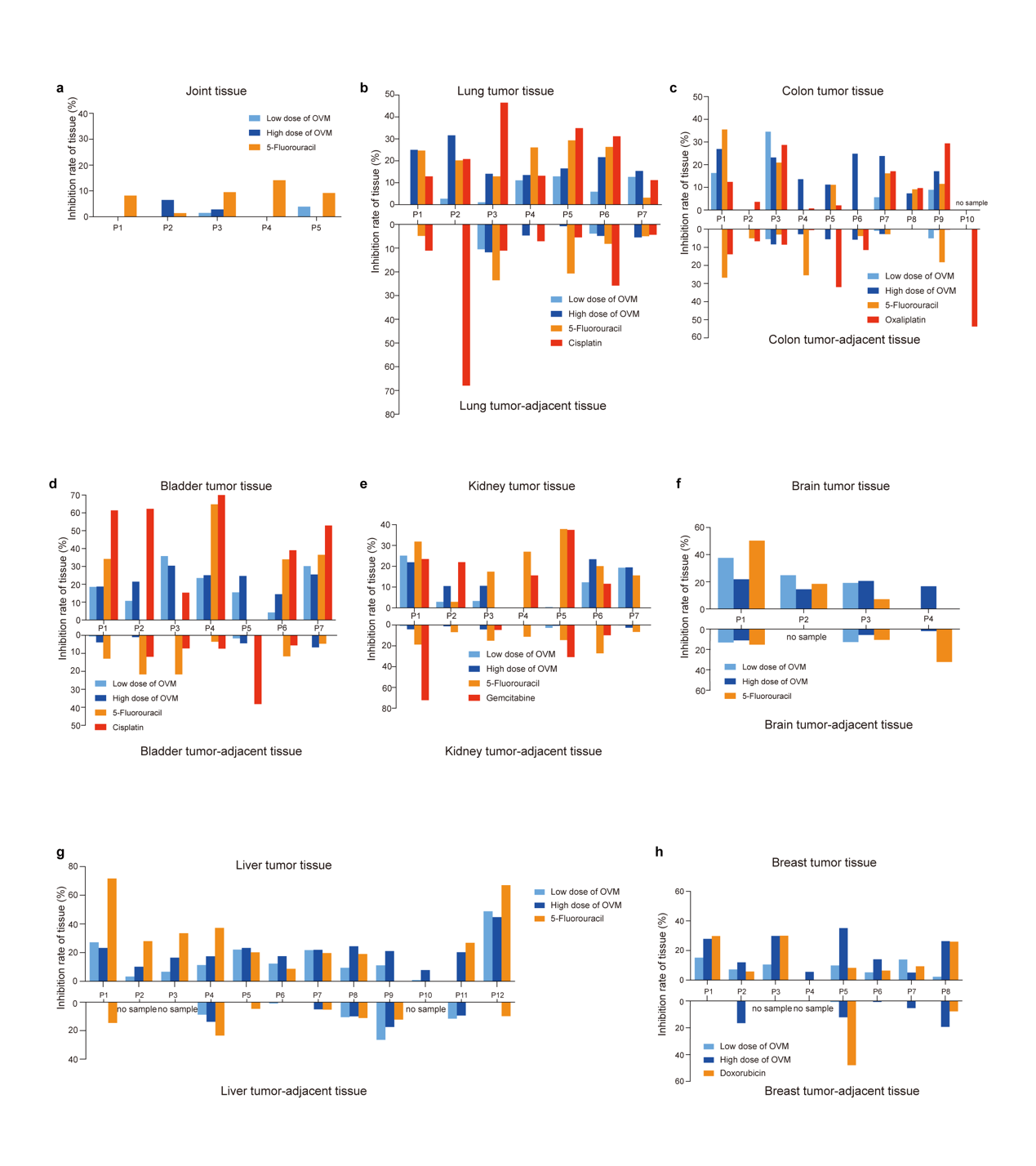
**

**Figure S9. Inhibition rate of tumor tissue and tumor-adjacent tissue treated with OVM in every patient. a** Inhibition rate of joint tissue under low dose of OVM, high dose of OVM and 5-Fluorouracil. **b*-*h** Inhibition rate of tumor tissue and tumor-adjacent tissue under OVM, 5-Fluorouracil, Cisplatin, Oxaliplatin, Doxorubicin or Gemcitabine treatment in lung, colon, bladder, kidney, brain, liver and breast.

**Table S1. Ranked table of the top 20 differentially expressed genes in turquoise module.**

| Gene name | Mean connectivity | Subcellular location* |
| --- | --- | --- |
| PXDN | 284 | Extracelluar matrix |
| CYR61 | 280 | Secreted |
| EMILIN1 | 280 | Extracelluar matrix |
| HSPG2 | 276 | Basement membrane |
| CRIM1 | 270 | Membrane protein |
| LOXL3 | 268 | Nucleus or cytoplasm |
| SCG2 | 266 | Secreted |
| NGF | 266 | Secreted |
| FN1 | 266 | Extracelluar matrix |
| ACTG2 | 266 | Cytoskeleton |
| RP11 | 264 | Nucleus |
| COL6A3 | 258 | Extracelluar matrix |
| PLOD1 | 256 | Endoplasmic reticulum |
| CCDC80 | 250 | Extracelluar matrix |
| COL8A1 | 240 | Basement membrane |
| ARSJ | 228 | Secreted |
| LTBP1 | 232 | Extracelluar matrix |
| MXRA8 | 212 | Membrane protein or nucleus |
| VCAN | 210 | Extracelluar matrix |
| GBE1 | 208 | Cytosol or cytoplasm |

* Subcellular location of candidate genes was annotated according to Uniprot database (https://beta.uniprot.org)

**Table S2. Cross correlation values between MXRA8 model and four MXRA8 densities of OVM-MXRA8 complex.**

| MXRA8 domain* | Site I | Site II | Site III | Site IV |
| --- | --- | --- | --- | --- |
| D1 inward | 0.9342 | 0.9413 | 0.9424 | 0.9353 |
| D2 inward | 0.9222 | 0.9140 | 0.9184 | 0.9175 |

* Crystal structure of MXRA8 ectodomain (PDB: 6JO8) was used and low passed to 12Å.

**Table S3. Descriptive statistics analysis of MXRA8 expression in human cancers.**

| Cancer types | MXRA8 expression (TPM) | | | | | | | | Numbers of Patient |
| --- | --- | --- | --- | --- | --- | --- | --- | --- | --- |
|  | Minimum | Maximum | Range | Mean | Std. Deviati-on | Std. Error of Mean | Coefficient of variation (%) | |  |
| LAML | -0.7834 | 4.282 | 5.065 | 1.954 | 0.967 | 0.074 | | 49.50 | 173 |
| ACC | 0.2522 | 9.776 | 9.524 | 3.522 | 1.799 | 0.205 | | 51.06 | 77 |
| BLCA | 1.043 | 10.36 | 9.314 | 5.120 | 1.686 | 0.082 | | 32.93 | 426 |
| LGG | 1.522 | 7.214 | 5.692 | 4.530 | 0.897 | 0.039 | | 19.81 | 522 |
| BRCA | 2.015 | 11.32 | 9.303 | 6.627 | 1.249 | 0.033 | | 18.84 | 1424 |
| CESC | -0.4325 | 8.383 | 8.815 | 4.468 | 1.422 | 0.082 | | 31.83 | 304 |
| CHOL | 2.141 | 7.770 | 5.629 | 5.228 | 1.110 | 0.185 | | 21.23 | 36 |
| COAD | 1.311 | 7.825 | 6.514 | 4.894 | 1.331 | 0.078 | | 27.19 | 288 |
| ESCA | 1.311 | 7.825 | 6.514 | 4.891 | 1.326 | 0.078 | | 27.11 | 286 |
| GBM | 3.052 | 8.967 | 5.915 | 5.558 | 1.047 | 0.081 | | 18.83 | 165 |
| KICH | -0.5543 | 9.539 | 10.09 | 2.091 | 1.509 | 0.186 | | 72.17 | 66 |
| KIRP | -0.6193 | 8.303 | 8.922 | 4.926 | 1.753 | 0.103 | | 35.59 | 288 |
| LIHC | -1.318 | 9.231 | 10.55 | 2.625 | 1.667 | 0.086 | | 63.53 | 369 |
| LUSC | 1.022 | 8.834 | 7.811 | 5.312 | 1.240 | 0.056 | | 23.35 | 498 |
| DLBC | 1.542 | 6.375 | 4.834 | 3.848 | 1.093 | 0.159 | | 28.40 | 47 |
| MESO | 2 | 8.648 | 6.648 | 6.036 | 1.396 | 0.150 | | 23.12 | 87 |
| OV | 0.6969 | 9.474 | 8.777 | 5.346 | 1.672 | 0.081 | | 31.27 | 426 |
| PAAD | 2.263 | 9.417 | 7.153 | 7.116 | 1.262 | 0.095 | | 17.73 | 178 |
| PCPG | -1.994 | 8.629 | 10.62 | 4.259 | 1.148 | 0.084 | | 26.96 | 185 |
| PRAD | 3.088 | 8.229 | 5.142 | 5.149 | 0.786 | 0.035 | | 15.27 | 495 |
| READ | 2.395 | 8.048 | 5.653 | 5.001 | 1.250 | 0.131 | | 24.99 | 91 |
| SARC | -0.0725 | 11.69 | 11.77 | 7.44 | 2.078 | 0.128 | | 27.93 | 264 |
| SKCM | -0.5332 | 9.943 | 10.48 | 4.284 | 1.608 | 0.074 | | 37.54 | 470 |
| STAD | 2.095 | 9.586 | 7.491 | 5.517 | 1.383 | 0.068 | | 25.07 | 414 |
| TGCT | 1.001 | 8.867 | 7.866 | 4.648 | 1.770 | 0.151 | | 38.08 | 137 |
| THYM | -0.2328 | 9.762 | 9.995 | 4.489 | 2.145 | 0.195 | | 47.78 | 121 |
| UCS | 0.542 | 8.567 | 8.025 | 4.826 | 1.401 | 0.104 | | 29.02 | 180 |
| UCEC | 0.542 | 8.567 | 8.025 | 4.826 | 1.401 | 0.104 | | 29.02 | 180 |
| UVM | 0.044 | 7.809 | 7.765 | 3.246 | 1.813 | 0.204 | | 55.84 | 79 |
| HNSC | 0.7664 | 10.35 | 9.583 | 5.014 | 1.381 | 0.061 | | 27.55 | 518 |
| KIRC | -2.114 | 9.946 | 12.06 | 5.250 | 1.650 | 0.072 | | 31.42 | 530 |
| LUAD | 2.248 | 9.695 | 7.447 | 6.073 | 1.228 | 0.054 | | 20.22 | 513 |
| THCA | 1.39 | 9.604 | 8.214 | 7.416 | 1.667 | 0.074 | | 22.48 | 504 |

**Table S4. Inhibition rates of patient derived primary tissues.**

| Tissue type | Drugs | Tissue inhibition rate (%) | | Patient number |
| --- | --- | --- | --- | --- |
|  |  | Tumor | Tumor-adjacent |  |
| Joint | Low dose OVM |  | 1.08 | 5 |
|  | High dose OVM |  | 1.86 |  |
|  | 5-Fluorouracil |  | 8.48 |  |
|  |  |  |  |  |
| Lung | Low dose OVM | 6.70 | 1.81 | 8 |
|  | High dose OVM | 19.60 | 3.98 |  |
|  | 5-Fluorouracil | 17.84 | 8.51 |  |
|  | Cisplatin | 24.39 | 19.00 |  |
|  |  |  |  |  |
| Colon | Low dose OVM | 6.53 | 1.15 | 10 |
|  | High dose OVM | 14.79 | 2.57 |  |
|  | 5-Fluorouracil | 10.43 | 8.53 |  |
|  | Oxaliplatin | 10.36 | 12.73 |  |
|  |  |  |  |  |
| Bladder | Low dose OVM | 19.77 | 0.33 | 7 |
|  | High dose OVM | 22.90 | 2.89 |  |
|  | 5-Fluorouracil | 24.21 | 10.91 |  |
|  | Oxaliplatin | 43.4 | 10.06 |  |
|  |  |  |  |  |
| Brain | Low dose OVM | 27.17 | 4.33 | 4 |
|  | High dose OVM | 18.43 | 3.17 |  |
|  | 5-Fluorouracil | 19.00 | 9.73 |  |
|  |  |  |  |  |
| Breast | Low dose OVM | 8.03 | 0.07 | 8 |
|  | High dose OVM | 19.50 | 4.53 |  |
|  | Doxorubicin | 14.45 | 4.65 |  |
|  |  |  |  |  |
| Liver | Low dose OVM | 14.61 | 6.50 | 12 |
|  | High dose OVM | 20.72 | 6.20 |  |
|  | 5-Fluorouracil | 28.43 | 9.04 |  |
|  |  |  |  |  |
| Kidney | Low dose OVM | 9.03 | 0.59 | 7 |
|  | High dose OVM | 12.27 | 1.86 |  |
|  | 5-Fluorouracil | 21.83 | 14.36 |  |
|  | Gemcitabine | 18.37 | 19.65 |  |

**References**

1. Zhang R*, et al.* Mxra8 is a receptor for multiple arthritogenic alphaviruses. *Nature*. **557**, 570-+ (2018).

2. Lin Y*, et al.* Identification and characterization of alphavirus M1 as a selective oncolytic virus targeting ZAP-defective human cancers. *Proc Natl Acad Sci U S A*.**111**, E4504-4512 (2014).

3. Zhang K. Gctf: Real-time CTF determination and correction. *J Struct Biol* . **193**, 1-12 (2016).

4. Tang G*, et al.* EMAN2: an extensible image processing suite for electron microscopy. *J Struct Biol*. **157**, 38-46 (2007).

5. Scheres SH, Chen S. Prevention of overfitting in cryo-EM structure determination. *Nat Methods*. **9**, 853-854 (2012).

6. Henderson R*, et al.* Outcome of the First Electron Microscopy Validation Task Force Meeting. *Structure*. **20**, 205-214 (2012).

7. van Heel M, Schatz M. Fourier shell correlation threshold criteria. *Journal of Structural Biology*. **151**, 250-262 (2005).

8. Pettersen EF*, et al.* UCSF Chimera-A visualization system for exploratory research and analysis. *Journal of Computational Chemistry*. **25**, 1605-1612 (2004).

9. Lee BSY, Jeon DG, Cho WH, Song WS, Kim MB, Park JH. Preliminary study of chemosenstivity tests in osteosarcoma using a histoculture drug response assay. *Anticancer Research*. **26**, 2929-2932 (2006).

10. Robert X, Gouet P. Deciphering key features in protein structures with the new ENDscript server. *Nucleic Acids Res*. **42**, W320-324 (2014).
